# Moving Morality Beyond the In-Group: Liberals and Conservatives Show Differences on Group-Framed Moral Foundations and These Differences Mediate the Relationships to Perceived Bias and Threat

**DOI:** 10.3389/fpsyg.2021.579908

**Published:** 2021-04-21

**Authors:** Brandon D. Stewart, David S. M. Morris

**Affiliations:** Department of Psychology, University of Birmingham, Birmingham, United Kingdom

**Keywords:** moral foundations, ingroups, political ideology, bias, threat, immigrants

## Abstract

Moral foundations research suggests that liberals care about moral values related to individual rights such as harm and fairness, while conservatives care about those foundations in addition to caring more about group rights such as loyalty, authority, and purity. However, the question remains about how conservatives and liberals differ in relation to group-level moral principles. We used two versions of the moral foundations questionnaire with the target group being either abstract or specific ingroups or outgroups. Across three studies, we observed that liberals showed more endorsement of Individualizing foundations (Harm and Fairness foundations) with an outgroup target, while conservatives showed more endorsement of Binding foundations (Loyalty, Authority, and Purity foundations) with an ingroup target. This general pattern was found when the framed, target-group was abstract (i.e., ‘ingroups’ and ‘outgroups’ in Study 1) and when target groups were specified about a general British-ingroup and an immigrant-outgroup (Studies 2 and 3). In Studies 2 and 3, both Individualizing-Ingroup Preference and Binding-Ingroup Preference scores predicted more Attitude Bias and more Negative Attitude Bias toward immigrants (Studies 2 and 3), more Implicit Bias (Study 3), and more Perceived Threat from immigrants (Studies 2 and 3). We also demonstrated that increasing liberalism was associated with less Attitude Bias and less Negative Bias toward immigrants (Studies 2 and 3), less Implicit Bias (Study 3), and less Perceived Threat from immigrants (Studies 2 and 3). Outgroup-individualizing foundations and Ingroup-Binding foundations showed different patterns of mediation of these effects.

## Introduction

To understand how people make sense of right and wrong in their social environment, Moral Foundations Theory proposed that five core moral values evolved to help direct social decisions and judgments (Haidt, 2012; Koleva et al., 2012). These moral foundations are Harm (e.g., decisions that hurt others), Fairness (e.g., giving everyone an equal chance), Loyalty (e.g., loyalty to a country or social group), Authority (e.g., respect for leaders, group roles, etc.), and Purity (e.g., cleanliness and religious sanctification; Haidt, 2007, 2012). Evidence has supported the idea that political liberals care about the moral foundations of Harm and Fairness most strongly, while conservatives care about the moral foundations of Loyalty, Authority, and Purity in addition to Harm and Fairness (Graham et al., 2009, 2011, 2012; Haidt, 2012; Koleva et al., 2012). However, the question arises of whether this differentiation is fully accurate and under what conditions it may be accurate or inaccurate. Other important questions regarding this distinction involve how moral foundations relate to interpersonal and intergroup processes. A deeper understanding of these relationships can help improve the dialog and communication between people with different political orientations when they discuss issues related to intergroup processes (e.g., immigrants and policies related to those issues).

### Individual Versus Group-Based Thinking

In considering differences in moral values between liberals and conservatives, researchers have suggested that the foundations of Harm and Fairness concern thinking about the effect on individuals and individual welfare. As liberals tend to endorse Harm and Fairness more strongly, it has been said that liberals tend to use *Individualizing* moral foundations that are concerned with the rights of individuals (Graham et al., 2009, 2011; Van Leeuwen and Park, 2009; Haidt, 2012). In contrast, conservatives may react more strongly to hearing about somebody who went against group principles, leaders, or beliefs (Loyalty, Authority, and Purity foundations), which are considered *Binding* foundations because they serve to bind groups together and the group is the focus of moral values and judgments (Graham et al., 2009, 2011; Van Leeuwen and Park, 2009). However, the findings by Graham et al. (2009, 2011) that conservatives endorse binding foundations more than liberals raises further questions in regard to morality and group-focused cognition, which have not been addressed by the current Moral Foundations Questionnaire (Graham et al., 2008; Voelkel and Brandt, 2019). This is due to the MFQ being ambiguous and referencing ingroups on only a small percentage of items. Questions also remain about whether liberals would demonstrate group-based moral concerns. If so, what types of groups may lead people to be more or less influenced? Finally, questions remain as to how distinctions in moral endorsement impact upon the broader intergroup context.

There are existing questions regarding group level and individualizing (harm and fairness) foundations and binding (loyalty, authority, and purity) foundations and whether there is an ingroup–outgroup distinction that may be obscured by the current framing of the moral foundations questions (Graham et al., 2008, 2012). In its current form, it is possible that some people will answer the questions on the widely used Moral Foundations Questionnaire (MFQ) by thinking about “society” or a generic “someone” in terms of their default or most important groups (e.g., their ingroups) while other people may think of a larger range of groups. Because the target group is vague within the moral foundations questionnaire and specified in only 17% of the items^1^, the groups that come to mind may be quite varied; this lack of clarity can make it difficult to interpret group effects on the MFQ. Understanding these group effects can also help explain the relationship between political ideology and intergroup judgments.

### Ideology and Intergroup Thinking

While there is ambiguity regarding moral foundations and the target group, work in other areas has highlighted the importance of processing distinctions in political ideology that should impact upon moral foundations, ideology, and intergroup processes. Hibbing et al. (2014) suggest that conservatives may be more vigilant to negativity, while others emphasize them as being more vigilant to threat cues (Lilienfeld and Latzman, 2014). Thus, in an intergroup context, conservatives compared to liberals may show ingroup over outgroup emphasis because of a wish to minimize risk to their ingroup. Other researchers have also noted the need for more appreciation of the impact of the group-level influence in moral foundations theory (Graham, 2013; Janoff-Bulman and Carnes, 2013; Talaifar and Swann, 2019). While researchers have expressed a need for investigating group-level influence, there has been little empirical evidence in moral foundations research and just one foundation within the Moral Foundations Questionnaire (MFQ) focuses on ingroup loyalty (Graham et al., 2011; Janoff-Bulman and Carnes, 2013, 2016; Smith et al., 2014; Voelkel and Brandt, 2019). Some evidence, however, has begun to show that manipulating a single foundation within the MFQ can influence endorsement. This research has shown that changing the authority figure to be either a liberal- or conservative-authority changes endorsement in relation to ideology so that liberals endorse the authority foundation more after liberal authorities and conservatives more after conservative authorities (Frimer et al., 2014). Importantly, the conservative authorities within this research represented groups that were all high in status within society (CEOs, Police Officers, Office Managers, Judges, Presidents, Traditions, and the Law), so they do not experience the same type of disadvantages as low status groups. Similar work has shown that manipulating the target of the purity judgment can lead liberals and conservatives to change their endorsement of purity (Frimer et al., 2015). Finally, very recent work has shown that liberals and conservatives endorse moral foundations more when the target groups are liberal or conservative; this research, however, did not investigate either a more general ingroup, or abstract-ingroups and abstract-outgroups (Voelkel and Brandt, 2019). Overall, these findings were important advancements, but they were limited to single moral foundations, or did not include abstract categorizations or low status groups.

Our approach focuses more broadly on groups and it focuses on ingroups and outgroups across all five moral foundations and uses both abstract and specific groups as targets. In addition, our work aims to have a strong applied focus in understanding immigration perceptions and perceptions toward a more neutral or general ingroup (i.e., people from Britain). We use a novel method of manipulating ingroup and outgroup targets of moral judgments by framing all of the items across the Moral Foundations Questionnaire for ingroups and outgroups at both the abstract- and specific-group level. We then use ideology to predict differences in endorsement of the group-focused foundations and to predict influences on attitudes, threat perceptions, and implicit bias toward immigrants. Detecting differences across the foundations by group framing provides important information regarding group-focused moral values by ideology, which has implications for Moral Foundations Theory. In addition, these differences may also inform attitudes and perceptions of immigrant groups and lead to potential avenues for dialogs. We therefore argue that making the target groups explicit within the Moral Foundations Questionnaire (MFQ) items in terms of outgroups and more general ingroups will reduce this ambiguity in the MFQ and provide a clearer picture of the relationship of political ideology to group-level processing in moral reasoning and to attitudes toward immigrants.

### Ingroups, Outgroups, and Moral Foundations

The current Moral Foundations Questionnaire is ambiguous and does not specify target groups beyond a few items. If all of the items in the MFQ were framed to identify abstract-ingroup and abstract-outgroup targets, then new differences may be detected between liberals and conservatives. Existing evidence suggests that liberals may show less endorsement and conservatives more endorsement if foundations are framed with an ingroup-target, with either abstract- or specific-ingroup, because of conservatives wanting to avoid threats and wanting to protect group boundaries, and liberals being more accepting of risk, more open to experience, and more promotion oriented (Jost et al., 2017). Research indicates that conservatives have demonstrated a tendency to be more vigilant to threats to their group, show more prevention motivation, more physiological reactivity in response to threatening stimuli, and have stronger motives too maintain social order (Federico et al., 2009; Carraro et al., 2011; Sibley et al., 2012; Janoff-Bulman and Carnes, 2013; Jost et al., 2017). Evidence of this promotion difference is observed in research showing that conservatives use more caution-based, avoidance techniques when investigating novel stimuli (Shook and Fazio, 2009; Cornwell and Higgins, 2013); in doing so, they were less susceptible to risks. Additional research has shown that conservatives endorse social order motives (e.g., adherence to appropriate behaviors and traditions) more than liberals, and liberals endorse social justice (e.g., protecting groups that are worse off) across a wider range of groups (Janoff-Bulman and Carnes, 2016). Overall, liberal’s focus on social justice equality and their acceptance of change are key differences that could contribute to them being more willing to account for low-status outgroups, such as immigrant groups, in their moral judgments (Duckitt, 2001; Jost et al., 2003; Jost, 2006; Duckitt and Sibley, 2009; Federico et al., 2009; Lenkeit et al., 2015; Portes, 2015). Thus, we would expect that conservatives would show more endorsement of moral foundations when they were framed about abstract-ingroups compared to abstract-outgroups than would liberals because of conservatives’ reduced tendency to be approach-motivated, to be less open to experience, and to be less focused on equality. This will be observed in a Higher Ingroup Preference Score in which higher scores indicate more endorsement of a moral foundation when framed about the ingroup (e.g., Binding-Ingroup Preference Score = Ingroup Binding minus Outgroup Binding scores). We would predict this to be the case for all five moral foundations and for the Individualizing and Binding composites (Itemized later in the Predictions section as Hypotheses 1a and 1b).

Our research will be the first to test the relationship of political ideology to the moral foundations questionnaire framed about abstract-groups and specific-groups (relating to immigrants). We believe the research associating liberalism with more approach orientation, more acceptance of change, and more focus on social equality suggests that liberals would be more likely to endorse harm reduction and fairness in relation to low-status outgroups such as immigrants (i.e., liberals show more endorsement of Outgroup-Individualizing foundations than conservatives; itemized later in the Predictions as Hypothesis 2b). We further predicted that liberals and conservatives would equally endorse harm reduction and fairness when framed about the abstract-ingroup because humans evolved in groups, so ingroups are important for all people and researchers advocate harm as a universal factor when making moral judgments (i.e., Ingroup-Individualizing foundations Hypothesis 2a; Brewer, 1999; Gray et al., 2012; Haidt, 2012). We would also predict that more conservatism will be related to more endorsement of *binding* concerns (loyalty, authority, and purity) when framed about an ingroup target because conservatives are more invested in avoiding risk and threats to the ingroup and more invested in social order, which are closely linked to loyalty, authority, and purity judgments (i.e., Ingroup-Binding foundations Hypothesis 2c). We did not expect either liberals or conservatives to care if people showed loyalty and respect for authority for the other group (i.e., Outgroup-Binding foundations Hypothesis 2d).

### Attitudes and Threat

If liberalism and conservatism relate differently to the ingroup–outgroup manipulation of targets of the moral foundations, we would predict that these differences could help explain differences in political ideology within an intergroup context (Van Leeuwen and Park, 2009). Threat-related motivation and goals of maintaining group and societal order have been demonstrated among those with more politically conservative ideologies (Duckitt, 2001; Jost et al., 2003; Jost, 2006; Duckitt and Sibley, 2009; Federico et al., 2009; Hibbing et al., 2014). These prevention-oriented motivations often appear to focus on social order, which may help with social coordination and protecting one’s groups from harm (Janoff-Bulman and Carnes, 2013, 2016). Researchers have demonstrated that a variety of threats, from threats to one’s self or one’s group, and threats to their country’s systems cause participants to show more self-reported conservatism and issue-based conservatism (Jost et al., 2007; Inbar et al., 2009; Thórisdóttir and Jost, 2011; Jost and Amodio, 2012; Terrizzi et al., 2013). In Studies 2 and 3 of the current research, we will examine the relationship of political ideology to threat and to the ingroup–outgroup moral foundations manipulation. We will investigate whether the conservatism to threat relationship is mediated by their higher endorsement of Ingroup-Binding foundations and lower endorsement of Outgroup-Individualizing foundations, and more endorsement of Ingroup Preference (Itemized later as Hypotheses 5c, 6c, and 6d).

Research has also indicated that liberals tend to show more positive attitudes toward low-status groups and to outgroups in general and that perceived threat and attitudes are intertwined tightly (Riek et al., 2006; Jost et al., 2017). While there are several exceptions (Brandt et al., 2014), liberals have demonstrated more positive attitudes toward gays and lesbians, Muslim Americans, and low-status groups, immigrants, and show less hostility to outgroups (Cunningham et al., 2004; Nisbet and Shanahan, 2004; Luguri et al., 2012; Kugler et al., 2014; Webster et al., 2014; Baldner and Pierro, 2019; Stewart et al., 2019). Again, differential endorsement of Ingroup-Binding and Outgroup-Individualizing foundations, and overall ingroup preference, may explain some of these differences. In Studies 2 and 3, we examine this relationship between political ideology and attitudes toward immigrants, and seek to test whether moral foundations mediate these effects (Hypotheses 5a and 5c, and 6a and 6b).

### Research Overview

In study 1, we will rewrite each of the MFQ items to contain an abstract-target group by adding the reference “ingroup” or “outgroup” to each item to focus on how ingroups and outgroups should be treated (Graham et al., 2008, 2011). For example, a harm item is written in the following way: “Whether or not someone acted unfairly toward my ingroup (my outgroup).” Participants will complete the entire revised-MFQ twice with the “ingroup” designation for one version and the “outgroup” designation for the other. In Studies 2 and 3, we will examine the relationship of political ideology to threat and to attitude bias (Hypotheses 5a to 5c), and whether different emphases on using specific-ingroups and specific-outgroups in moral judgments mediate the relationships between political ideology and threat, and political ideology and attitudes toward immigrants (Hypotheses 6a to 6d). Investigating the use of outgroups and general ingroups when making moral judgments can be helpful in understanding differences in reactions to immigrants and other outgroups, and how to frame discussions that will likely continue given the need for immigration to offset low birth rates in the United Kingdom, the United States, and the world. These debates currently show a deep partisan divide in many countries and are important to a variety of topics related to intergroup contexts.

## Study 1 Predictions

Study 1 framed the moral foundations questionnaire in terms of the abstract-group level.

Hypotheses 1a and 1b: We hypothesized that liberals would have lower Ingroup Preference in comparison to conservatives.

• Liberals would show less endorsement of Individualizing (1a) and Binding foundations (1b) when framed about the Ingroup compared to the Outgroup (i.e., Individualizing-Ingroup Preference Score = Ingroup-harm and Ingroup-fairness composite minus the Outgroup-harm and Outgroup-fairness composite; the same calculation is used for the binding composite by using the Loyalty, Authority and Purity foundations).

Hypotheses 2a, 2b, 2c, and 2d: Examining the ingroup- and outgroup-foundations separately would clarify where the differences exist.

• We predicted that political ideology would not be significantly related to endorsement of the Ingroup-individualizing foundations (average of Ingroup-Harm and Ingroup-Fairness; 2a).
• We predicted that Liberals would endorse Outgroup-Individualizing foundations (outgroup-harm and -fairness) more than Conservatives (2b) because the literature indicates that liberals may have a group-based social justice orientation as well as more approach motivation (Carney et al., 2008; Shook and Fazio, 2009; Janoff-Bulman and Carnes, 2013).
• For the Binding foundations, we predicted that Conservatives would endorse Ingroup-Binding foundations (Ingroup-Loyalty, -Authority, and -Purity) more thank Liberals (2c), which is in accordance with conservatives’ focus on ingroups (Graham et al., 2009; Shook and Fazio, 2009; Kugler et al., 2014).
• For the Outgroup-Binding foundations (Outgroup-Loyalty, -Authority, and –Purity), we predicted that no differences will be detected (2d).

### Method

#### Participants and Design

Any participant demonstrating inattention on the moral foundations questionnaires (MFQ) was excluded from analyses as recommended by Graham et al. (2009, 2012; Haidt, 2007; see Supplementary Table 1 for distributions for all three studies). An example of this inattention is answering that it is more than slightly relevant that someone is good at mathematics “when you decide something is right or wrong.” Our final sample therefore consisted of 153 participants^2^ from the University of Birmingham (United Kingdom) with an age range of 18–35 years (*M* = 20.57, *SD* = 2.66), 78.4% were White, and 49.0% were Liberal, 35.3% were Moderate and 15.7% were Conservative; this is a similar range to the one observed in the Graham et al. (2009) studies. The study used a within-participants manipulation of group focus (participants completed both the ingroup- and the outgroup-versions of the MFQ), and political orientation was a continuous predictor. To statistically control for order effects, we counterbalanced the presentation of the ingroup MFQ and outgroup MFQ measures.

#### Materials and Procedure

##### Group-framed moral foundations (MFQ)

Participants provided informed consent and then were randomly assigned to receive either the ingroup-MFQ first or the outgroup-MFQ first order (i.e., ingroup–outgroup manipulation). Participants read a description explaining the meaning of either ingroups or outgroups [see Supplementary Materials section “Study 1: The (Abstract) Ingroup and Outgroup Framed MFQs”]. The ingroup version stated that: “*In this section of the study we will ask you to think about “INGROUPS.” For the purposes of this study, an INGROUP is any group or groups of which you DO class yourself as being a member, or belonging to, and that you identify with.*” In the outgroup version, participants read an identical description, but instead about an “OUTGROUP” (Brewer and Brown, 1998). We altered each of the 30 items to be about either an Ingroup for the ingroup-MFQ or an Outgroup for the outgroup-MFQ (see Table 1 for example items). Our focus was on systematically varying the target group, and we did not deviate from this procedure on any single item. This focus on systematic changes, unfortunately, did allow some ambiguity to remain and we discuss this issue in the general discussion. For data analysis in all studies, the computer program coded the relevance subscales of the MFQ as 1 = Not at all relevant, and 6 = Extremely relevant, and the judgment subscales were coded 1 = Strongly disagree, and 6 = Strongly agree (see Supplementary Table 1 section “Reliability Analyses” for reliability data for all three studies).

**TABLE 1:** Moral Foundations Questionnaire framed about either an Ingroup or an Outgroup.

**MFQ version**	**Item wording**
Ingroup item 1	Whether or not someone in my ingroup suffered emotionally.
Ingroup item 2	Whether or not some people from my ingroup were treated differently than others.
Ingroup item 3	Whether or not someone’s action showed love for his or her country, which is also my ingroup.
Ingroup item 4	Whether or not someone showed a lack of respect for authority of my ingroup.
Ingroup item 5	Whether or not someone violated my ingroup’s standards of purity and decency.
Outgroup item 1	Whether or not someone in an outgroup suffered emotionally.
Outgroup item 2	Whether or not some people from an outgroup were treated differently than others.
Outgroup item 3	Whether or not someone’s action showed love for his or her country, which is also an outgroup to my country.
Outgroup item 4	Whether or not someone showed a lack of respect for authority of an outgroup.
Outgroup item 5	Whether or not someone violated an outgroup’s standards of purity and decency.

##### Filler task

After completing the first MFQ, participants completed a short, filler-task of cognitive processing that separated the two MFQs. The filler lasted for 40 trials with each trial asking participants to select a target number as fast as possible among 9 competing distractor numbers (10 items per set). After completing the filler task, participants completed the version of the MFQ that they had not yet completed.

##### Demographics and political ideology

Once both versions of the MFQ had been completed, a series of measures, unrelated to the current study, were completed^3^ and were followed by questions about demographics. Self-rated political ideology (Jost, 2006) was included among the demographic questions on age, gender, race, national identity, intergroup ideology, left-right political ideology, English as a second language, and years lived in the United Kingdom. The political ideology item was adapted from Jost and colleauges (Jost, 2006; Jost et al., 2007) and consisted of a nine-point, vertical scale where participants were asked to: “Please rate your, personal political orientation” ranging from, at the top, 1 “*Extremely Conservative”* to 9 “*Extremely Liberal,”* with a midpoint of 5 “*Center/Moderate.*” This item has been used in previous moral foundations research in the United States and United Kingdom and is considered stable and is frequently included at the end of the study (Carney et al., 2008; Graham et al., 2009; Terrizzi et al., 2010; Krosch et al., 2013; Crawford et al., 2015; Janoff-Bulman and Carnes, 2016). All participants were fully debriefed upon study completion.

### Results

#### Ingroup Preference

We first tested Hypotheses 1a and 1b. For the Individualizing and Binding groupings, a linear regression was conducted with Political Ideology as the predictor and Ingroup Preference Score as the outcome. As an example, Individualizing-Ingroup Preference equals ingroup-Individualizing minus outgroup-Individualizing scores. For Political Ideology, higher scores indicated higher liberalism and lower scores indicated more conservatism (e.g., Individualizing-Ingroup Preference = ingroup-harm minus outgroup-harm; Hypothesis 1a). Higher Ingroup Preference Scores would indicate more endorsement of the moral foundation when it was framed about the ingroup. A negative regression coefficient between Ingroup Preference Score and Political Ideology indicated that conservatives showed more endorsement and liberals less endorsement of the moral foundation when it was framed about the ingroup as opposed to an outgroup and demonstrated the effect of the framing manipulation on endorsement by liberals and conservatives (Judd, 2000). As predicted, we observed that more liberalism was related to less Individualizing- and less Binding-Ingroup Preference (i.e., Negative relationship supports Hypotheses 1a and 1b; see Table 2; see Supplementary Materials sections “Ingroup Preference Regressions for Each of the Five Foundations for Studies 1, 2, and 3” and “Ingroup and Outgroup Regressions for Each of the Five Foundations for Studies 1, 2, and 3.” for analyses of the five individual foundations). Detecting these differences showed the effectiveness of the ingroup versus outgroup framing of moral foundations.

**TABLE 2:** Standardized regression coefficients (β) for separate regression equations with political ideology predicting each framed moral foundation ingroup-preference score separately.

	**Political ideology**					
	**Separate linear regressions**				**Bootstrapping (BCa)**	
	**β**	***p*-value**	***R*^2^**	**df**	***b***	**95% CI for b**

Individualizing-ingroup preference	−0.31***	<0.001	0.10	151	−0.10	[−0.155, −0.042 ]
Binding-ingroup preference	−0.41***	<0.001	0.17	151	−0.15	[−0.210, −0.091]

Higher scores on ideology reflected increased liberalism (vs. conservatism). Higher Ingroup Preference Scores indicated more investment in the moral foundation when it was framed about the ingroup. A negative regression coefficient between Preference Score and Political Ideology indicated that conservatives showed more endorsement of the moral foundation when it was framed about the ingroup as opposed to the outgroup. Bootstrapping analyses were conducted to 5,000 samples. ***p < 0.001.

#### Separate Ingroup- and Outgroup-Framed Foundations

In order to parse the data by group-framing, we conducted a series of individual linear regressions for Political Ideology and its relationship to the Ingroup- and Outgroup-Individualizing and the Ingroup- and Outgroup-Binding Composites (see Table 3). This allowed for a more nuanced analysis in which we could check if the differences were in the Ingroup-version or the Outgroup-version of the MFQ. As expected, we found that political ideology did not relate significantly to Ingroup-Individualizing Foundations (Average of Ingroup-Harm and Ingroup-Fairness; Hypothesis 2a), but it did significantly relate to Ingroup-Binding Foundations (Average of Ingroup-Loyalty, Ingroup-Authority, and Ingroup-Purity; Hypothesis 2c); this effect showed that liberals were significantly less invested in the Ingroup-Binding foundations than were conservatives. Also, as expected, we observed that more liberalism was related to significantly more endorsement of Outgroup-Individualizing foundations (Hypothesis 2b). Unexpectedly, more conservatism was significantly related to more endorsement of Outgroup-Binding, which had not been predicted (Hypothesis 2d). See Supplementary Materials Section “Graphs of the Individualizing and Binding Foundations for Ingroups and Outgroups” for the graphs of the regressions for individualizing and binding foundations and political orientation analyses. (see also Supplementary Materials sections “Ingroup Preference Regressions for Each of the Five Foundations for Studies 1, 2, and 3” and “Ingroup and Outgroup Regressions for Each of the Five Foundations for Studies 1, 2, and 3” for analyses related to the individual foundations).

**TABLE 3:** Four linear regressions with political ideology as the predictor and each ingroup and outgroup moral foundation entered as a separate outcome measure.

	**Political ideology**					
	**Separate linear regressions**				**Bootstrapping (BCa)**	
	**β**	***p*-value**	***R*^2^**	**df**	***b***	**95% CI for *b***

**Ingroup referent**						
Ingroup-individualizing	0.01	=0.870	0.00	151	0.01	[−0.056, 0.060]
Ingroup-binding	−0.52***	<0.001	0.27	151	−0.26	[−0.331, −0.196]
**Outgroup referent**						
Outgroup-individualizing	0.24**	=0.003	0.06	151	0.10	[0.031, 0.174]
Outgroup-binding	−0.25**	=0.002	0.06	151	−0.12	[−0.188, −0.043]

*MFQ variables were re-scored so that they matched the 0–5 coding for the Moral Foundations Questionnaire*. **p < 0.01, ***p < 0.001.

### Discussion

This study demonstrated that framing moral foundations to be about either an abstract-ingroup or abstract-outgroup altered the foundation endorsement of liberals and conservatives (Graham et al., 2009, 2011; Haidt, 2012; Frimer et al., 2014). The findings for the Ingroup-Preference Scores are the first to show that changes in MFQ-endorsement were due to the manipulation of an abstract-ingroup or abstract-outgroup in the Moral Foundations questionnaire. Ingroup preference was significantly less for liberals than conservatives for both Individualizing and Binding foundations.

Overall, these results indicated that liberals and conservatives differed in their endorsement based upon groups. The results were significant across Individualizing and Binding foundations as opposed to just one foundation or only to specific, high status groups. In order to establish where the differences in preference scores lay, we examined the ingroup- and outgroup-framed foundations. As predicted, when we considered the relationship between each of the ingroup and then the outgroup foundations and ideology, there was no difference between liberals and conservatives for Ingroup-Individualizing foundations, but conservatives endorsed the Ingroup-Binding foundations more (Ingroup-Loyalty, Ingroup-Authority, and Ingroup-Purity as a composite). As predicted, liberals endorsed the Outgroup-Individualizing foundations more than did conservatives, but unexpectedly, conservatives were more invested in Outgroup-Binding foundations. In Study 2, we sought to replicate these effects using a more representative, online sample. We also sought to rule-out whether liberals and conservatives thought about more positive or less positive groups when they considered abstract-ingroups and -outgroups because participants could think of any group they wanted. To accomplish this goal, we identified specific groups within the MFQ versions, which would be relevant to understanding broader intergroup relations and attitudes toward immigrants. We also sought to test whether endorsement of foundations when framed about ingroups and outgroups mediated the often-observed political ideology to negative immigrant attitudes relationship that has been observed in the literature. Because we manipulated ingroup and outgroup-MFQ focus and because moral foundations are proposed to be very fundamental motivations, we sought to use them as mediators here.

## Study 2

Study 1 demonstrated the advantages of considering the group level to gain a better understanding of the relationship between political ideology and morality and to add to the existing literature showing that the type of groups imagined do matter for all five foundations and not just individual foundations (Frimer et al., 2014, 2015). The observed differences of liberals and conservatives in the endorsement of judgments based upon abstract-ingroups and abstract-outgroups can also influence social cognition within an intergroup context. For example, these differences can be used to help explain political differences in attitudes and perceived threat from immigrants that have been routinely observed within the literature. It is also important to test whether the Study 1 findings were due to liberals and conservatives thinking of very different groups when answering the moral questions, which is a possibility when no limits are imposed on the MFQ or when the groups are abstract. In Study 2, we will use a version of the framed-MFQ that specifies British people as the ingroup and Pakistani immigrants as the outgroup. This framing will bolster the confidence that the results of Study 1 using abstract-ingroups and abstract-outgroups also relate to specific and real ingroups and outgroups within the context of immigration. We predict a replication of effects in which liberals would show less endorsement than conservatives for the foundations about the British ingroup in comparison to foundations framed about a Pakistani immigrant outgroup (Itemized later in the Predictions section as Hypotheses 3a and 3b). We used this outgroup because it is a large and rapidly growing minority group and we chose people from Britain as the ingroup because it should not be considered as either a conservative or a liberal authority because all participants belonged to the group (Voelkel and Brandt, 2019). Testing our effects with specific groups, as opposed to abstract groups, will also help to demonstrate the generality and robustness of the findings in relation to a specific ingroup and specific low-status outgroup (Koleva et al., 2012). We predict that the ingroup and outgroup effects seen with abstract-groups in Study 1 would be replicated with the specific-groups in Study 2. Thus, we hypothesize that more liberalism would be associated with more endorsement of Outgroup-Individualizing foundations (Hypothesis 4b) and less endorsement of Ingroup-Binding foundations (Hypothesis 4c). In Study 2, we will also use a sample of participants from the general community to broaden the representativeness of the results.

Based upon Study 1, more endorsement of fairness and reducing harm when outgroups are considered may be associated with reduced bias against different groups, and may explain liberal’s propensity to have more positive views of outgroups in general and in regard to ethnicity and immigration. While there are a number of exceptions (Brandt et al., 2014), liberals tend to show an acceptance of a wider range of outgroups in general and of low-status groups, including immigrants (Jost et al., 2017). Research has shown that liberals focus more on social equality, have more positive attitudes toward gays and lesbians, Muslim Americans, or Arabs, demonstrate less outgroup hostility, and show more positive feelings toward low-status groups and immigrants (Whitley and Lee, 2000; Duckitt, 2001; Jost et al., 2003, 2017; Cunningham et al., 2004; Nisbet and Shanahan, 2004; Duckitt and Sibley, 2009; Federico et al., 2009; Luguri et al., 2012; Kugler et al., 2014; Webster et al., 2014; Baldner and Pierro, 2019; Stewart et al., 2019). Thus, a major aim of Study 2 was to test the relationship between more liberalism and less negative attitudes toward immigrants (Hypotheses 5a and 5b) and between more liberalism and less perceived threat (Hypothesis 5c). Since the ingroup- and outgroup-MFQ targets were manipulated and because moral foundations are proposed to be fundamental motives, this aim also included testing that the endorsement of outgroup-individualizing foundations of Harm and Fairness would mediate the political effect on bias (Hypothesis 6a) and on threat (Hypothesis 6c) for a low status group. Whether the ingroup-binding (Ingroup-Loyalty, Ingroup-Authority, and Ingroup-Purity) foundations would also be related to attitude bias and mediate the political effect is much less clear because there is sparse evidence on the relationships of moral foundations and attitude bias (Hypothesis 6b). However, some previous work has shown that more endorsement of binding foundations was related to more negative attitudes toward immigrants and to more outgroup hostility (Kugler et al., 2014; Baldner and Pierro, 2019).

A third extension of our findings relates to explaining the differences in perceived threat responses to ethnic groups and immigrants. Previous research has shown that conservatives generally show more vigilance for threatening stimuli, more threat from unfamiliar groups, and that needs for threat management were associated with conservatism (Jost et al., 2007, 2017; Inbar et al., 2009; Dodd et al., 2012; Jost and Amodio, 2012; Hibbing et al., 2014). Given these findings, we were interested in examining whether ingroup binding foundations would predict perceptions of threat from immigrants, and that endorsement of ingroup binding foundations (i.e., composite of Ingroup-Loyalty, Ingroup-Authority, and Ingroup-Purity) would mediate the relationship of more conservatism predicting more perceived threat, potentially as a function of group boundary maintenance concerns (Hypothesis 6d).

### Predictions

Hypotheses 3a and 3b: We predicted a replication of the main pattern of associations for Individualizing-Ingroup Preference (3a) and Binding-Ingroup Preference (3b). Liberals would show less endorsement of Individualizing and Binding foundations when framed about the British ingroup compared a Pakistani immigrant outgroup.

Hypotheses 4a, 4b, 4c, and 4d: We predicted a replication of the Study 1 results. More liberalism would be related to more endorsement of Outgroup-Individualizing foundations (4b), while more conservatism would be related to more endorsement of Ingroup-Binding foundations (4c).

Hypotheses 5a through 5c: We predicted that more liberalism would be significantly related to less Attitude Bias, less Negative Bias, and less Perceived Threat from outgroups (5a, 5b, and 5c). Related to this prediction is the expectation that each Ingroup-Preference index would positively predict Attitude Bias, Negative Bias, and Threat. This prediction is supported by the intergroup bias literature, which has demonstrated that strong ingroup favoritism (i.e., similar to ingroup preference) is related to increased intergroup bias (Brewer, 1999; Buttelmann and Böhm, 2014; Greenwald and Pettigrew, 2014). Because of space considerations we have included these analyses within Table 1 of the Supplementary Materials (see section “Individualizing-Ingroup Preference to Attitude Bias, to Negative Bias, and to Threat Regressions, and Binding-Ingroup Preference to Attitude Bias, to Negative Bias, and to Threat regressions”).

Additionally, past research has not found a significant link between political ideology and cognitive perspective taking ability. Thus, we did not expect a significant relationship here (Jost et al., 2003; Falk et al., 2012).

### Mediation Hypotheses

Hypothesis 6a: Regarding attitude bias and negative bias, we predicted that a more liberal ideology would be associated with lower levels of bias toward immigrants, and that this relationship would be significantly mediated by more endorsement of the Outgroup-Individualizing foundations (average of Outgroup-Harm and Outgroup-Fairness).

Hypothesis 6b: We tentatively predicted that the more endorsement of Ingroup-Binding foundations would meditate the political ideology to attitude bias and to negative bias effects (Kugler et al., 2014).

Hypothesis 6c and 6d: We predicted that a liberal orientation would be associated with less perceived threat, and that this would be mediated by lower endorsement of British Ingroup-Binding foundations and by more endorsement of Outgroup-Individualizing foundations.

Because political ideology had to be measured, we could manipulate only the mediator to establish its causal influence and demonstrate the effectiveness of the manipulation (Spencer et al., 2005). Thus, in Studies 2 and 3, we treated Outgroup-Individualizing and Ingroup-Binding similar to measured mediators and we used the manipulated Individualizing-Ingroup Preference and manipulated Binding-Ingroup Preference scores to demonstrate the influence of the manipulation on the Outcomes (Attitude Bias, Negative Bias, and Threat). See Supplementary Section “Individualizing-Ingroup Preference to Attitude Bias, to Negative Bias, and to Threat Regressions, and Binding-Ingroup Preference to Attitude Bias, to Negative Bias, and to Threat regressions” for the analyses.

### Method

#### Participants and Design

We recruited participants from the United Kingdom using the Prolific.co online recruitment platform. Based upon Study 1 effect sizes and upon screening criteria for the moral foundations questionnaire and other online studies we have conducted using the questionnaire, we recruited three hundred and fifty participants to obtain a final sample close to 300 participants and to observe 0.8 to 0.85 power for a small to medium effect, *f*^2^ = 0.031/*R*^2^ = 0.03 (Woods et al., 2015). Prolific.co is a tool used to recruit participants in online settings and it is meant to provide a larger and more varied sample of participants than MTurk, which has been demonstrated to be an effective means of collecting data of comparable quality to laboratory data (Buhrmester et al., 2011; Casler et al., 2013; Paolacci and Chandler, 2014). In total, 351 participants completed the dependent variables, and we first removed four participants who were of Pakistani ethnicity and three people who had not been born in the United Kingdom (see Supplementary MaterialsTable 1 section “Distribution of Removals”). Next, we removed thirty-seven participants who showed inattention on the MFQs using the same criteria as in Study 1 (Graham et al., 2009). The study had a final sample of 307 participants, all of whom currently lived in the United Kingdom, and who had an age range of 18–80 years (*M* = 35.94, *SD* = 12.10); 51.8% were Liberal, 23.8% Moderate, and 24.4% were Conservative while 88.9% were White and 58.0% were Female.

#### Materials and Procedure

Participants completed informed consent and then were asked to select the letter that appeared at the top of the screen from a randomized list (i.e., random assignment), and then, on the next screen, to click the number 2 to ensure the data was writing.

##### Group-framed moral foundations questionnaire (MFQ)

The first part of the study used the same within-participants manipulation and procedure as Study 1 in which participants completed both the ingroup and outgroup versions of the moral foundations questionnaire with order counterbalanced. However, in Study 2, the two versions of the MFQ were now framed so that specific groups in the United Kingdom were referenced. For the ingroup-version, participants read about moral foundations framed about British people because it was a neutral ingroup. For the outgroup-version, the questions were framed about Pakistani Immigrants as the target group because this is sizable and growing group within the United Kingdom, and it reduces variance because it is very specific (see Supplementary section “Study 1: The (Abstract) Ingroup and Outgroup Framed MFQs”).

##### Filler task

Two versions of the MFQ were again separated with the same filler task from Study 1. Participants next received measures of attitude bias, negative bias, and then perspective taking and perception of threat from immigrants with the latter two measures being counterbalanced.

##### Attitude bias

The measure of attitude bias toward immigrants was adapted from Saguy et al. (2009). Participants rated their feelings toward Pakistani immigrants on five evaluative dimensions (i.e., Negativity, Friendliness, Warmth, Trusting, and Disgust) with a nine-point scale with endpoints from one dimension to the opposite dimension (e.g., “*extremely cold*” to “*extremely warm*”). After reverse scoring two items, they were averaged to create an index of bias with higher scores indicating higher levels of bias (α = 0.94).

##### Negative attitude bias

A second measure of negative bias was used to determine negative attitudes toward Pakistani immigrants and was adapted from Stephan et al. (2002). The scale included 5 items assessing levels of disapproval, resentment, dislike, disdain, and hatred; these items were completed on a 10-point scale with endpoints changing to reflect the construct being measured and scored from 0 “*no _____ at all” (e.g., no dislike at all) to* 9 *“Extreme” (e.g., Extreme dislike)*. These items were coded by the computer from 1 to 10 and had high reliability (α = 0.96).

##### Perspective taking

Participants were randomly assigned to complete either the Perspective Taking scale first and then the Threat scale, or vice versa. The seven item Perspective Taking subscale of the Interpersonal Reactivity Index assessed participants’ ability to perceive the world from the perspectives of others (Davis, 1983; α = 0.83).

##### Threat perceptions

Participants completed a measure of threat perceptions toward Pakistani immigrants in the United Kingdom, which was adapted from Stephan et al. (1999). This scale contained 15 items measuring attitudes toward both realistic threats (i.e., resource-based; 8 items) as well as symbolic threats (i.e., cultural beliefs and values; 7 items) from immigrant groups. All items were completed on a seven-point scale ranging from 1 “*Disagree Strongly*” to 7 “*Agree Strongly*” with “*Neither Agree nor Disagree*” as the neutral midpoint. After reverse scoring several items, the average represented an index of threat perceptions toward immigrants; reliability for the scale was high (α = 0.94). Because the subscales share a common theme of threats to the ingroup and because the correlation between symbolic and realistic threat was extremely high (*r* = 0.78, *p* < 0.001), we used the overall index of threat as has been done in previous research (Stephan et al., 1999; Verkuyten, 2009; Tip et al., 2012).

##### Demographics and political ideology

Finally, participants completed demographic measures that included two simple, mathematics filler problems, political ideology, their age and gender, questions about the purpose of the study and tasks being related, being born in the United Kingdom, race, country in which they lived, years living in the country, English as a second language, and whether they had been in the exact study previously.

### Results

#### Ingroup Preference

Separate, linear regressions were performed with Political Ideology as the predictor and Individualizing-Ingroup Preference and Binding-Ingroup Preference as separate Outcome measures (e.g., Individualizing Ingroup-Preference Score = Ingroup Individualizing minus Outgroup Individualizing foundations). The Ingroup Preference score demonstrated the effectiveness of the ingroup–outgroup framing manipulation on foundation endorsement as a function of political ideology (Hypotheses 3a and 3b). We observed that more liberalism was related to significantly less Individualizing-Ingroup Preference and less Binding-Ingroup Preference, replicating the framing manipulation’s impact on endorsement (see Table 4).

**TABLE 4:** Standardized regression coefficients (β) for separate regression equations with political ideology predicting each framed moral foundation ingroup-preference score separately.

	**Political ideology**					
	**Separate linear regressions**				**Bootstrapping (BCa)**	
	**β**	***p*-value**	***R*^2^**	**df**	***b***	**95% CI for *b***

Individualizing-ingroup preference	−0.24***	<0.001	0.06	305	−0.10	[−0.156, −0.051]
Binding-ingroup preference	−0.39***	<0.001	0.15	305	−0.16	[−0.208, −0.107]

Higher scores on ideology reflected increased liberalism (vs. conservatism). Higher Ingroup Preference Scores indicated more investment in the moral foundation when it was framed about the ingroup. A negative regression coefficient between Preference Score and Political Ideology indicated that conservatives showed more endorsement of the moral foundation when it was framed about the ingroup as opposed to the outgroup. Bootstrapping analyses were conducted to 5,000 samples. ***p < 0.001.

#### Separate Ingroup- and Outgroup-Framed Foundations

As expected, we observed that Political Ideology was not significantly related to the Ingroup-Individualizing composite (see Table 5; Hypothesis 4a). For Ingroup-Binding foundations, more liberalism was related to significantly less Ingroup-Binding endorsement as predicted (Hypothesis 4c). This result shows that conservatives showed more endorsement and liberals less endorsement of binding foundations when framed about the ingroup. Also, as predicted, more liberalism was related to significantly more Outgroup-Individualizing endorsement such that liberals were more invested than conservatives in Harm and Fairness when framed about the Pakistani-immigrant outgroup (Hypothesis 4b). In a replication of Study 1, we found that Political Ideology was significantly correlated with the Outgroup-Binding composite, though this was a very small effect (*R*^2^ = 0.016) and was non-significant with Bonferroni corrections for four comparisons (Hypothesis 4d). It is possible that when Binding foundations are about an outgroup and the outgroup is abstract, conservatives compared to liberals will want outgroup members to remain loyal to their group (outgroup), which could have increased conservatives’ endorsement of those foundations. Future research will need to disentangle these effects. See Supplementary Materials Section “Graphs of the Individualizing and Binding Foundations for Ingroups and Outgroups” for the regression graphs of the individualizing and binding foundations and political orientation analyses.

**TABLE 5:** Four linear regressions with political ideology as the predictor and each Ingroup and Outgroup moral foundation entered as a separate outcome measure.

	**Political ideology**					
	**Separate linear regressions**				**Bootstrapping (BCa)**	
	**β**	***p*-value**	***R*^2^**	**df**	***b***	**95% CI for *b***

**Ingroup referent**						
Ingroup-individualizing	0.08	=0.175	0.01	305	0.04	[−0.019, 0.090]
Ingroup-binding	−0.44***	<0.001	0.19	305	−0.21	[−0.265, −0.160]
**Outgroup referent**						
Outgroup-individualizing	0.29***	<0.001	0.08	305	0.14	[0.085, 0.195]
Outgroup-binding	−0.13*	=0.029	0.02	305	−0.05	[−0.103, −0.006]

*MFQ variables were re-scored so that they matched the 0–5 coding for the Moral Foundations Questionnaire*. *p < 0.05, ***p < 0.001.

#### Ideology to Attitudes and Threat

We next conducted three linear regressions to test the hypotheses that increased liberalism would be related to less Attitude Bias, less Negative Bias, less Perceived Threat from immigrants, and that Political Ideology would not be related to differences in Cognitive Perspective Taking; we use bias to mean response tendency instead of error. Once again, higher scores on Political Ideology indicated a more liberal ideology. As predicted, we observed that increasing liberalism was significantly related to less Bias, *R*^2^ = 0.15, β = −0.39, *t*(305) = −7.36, *p* < 0.001, as well as significantly less Negative Bias, *R*^2^ = 0.14, β = −0.37, *t*(305) = −6.95, *p* < 0.001, and significantly less Perceived Threat, *R*^2^ = 0.28, β = −0.53, *t*(305) = −10.91, *p* < 0.001 (Hypotheses 5a through 5c). Unexpectedly, Political Ideology was related to Cognitive Perspective Taking ability. *R*^2^ = 0.06, β = 0.25, *t*(305) = 4.44, *p* < 0.001. Next, we predicted that ingroup preference would be related to more bias because the literature has demonstrated that more ingroup favoritism is related to more intergroup bias. Supporting this prediction, we observed that more Individualizing-Ingroup Preference and more Binding-Ingroup Preference were significantly related to more Attitude Bias, Negative Attitude Bias, and more Threat; this result demonstrated the significant influence of the manipulated-mediators on the Bias and Threat outcome variables (see Supplementary section “Individualizing-Ingroup Preference to Attitude Bias, to Negative Bias, and to Threat Regressions, and Binding-Ingroup Preference to Attitude Bias, to Negative Bias, and to Threat regressions” for the regressions and discussion of these predictions). Finally, we have included a correlation table of the main measures to provide an overall picture of their relationships (see Table 6).

**TABLE 6:** Pearson correlations (*r*) between main study variables.

	**1**	**2**	**3**	**4**	**5**	**6**
(1) Political ideology	–	0.29***	−0.44***	−0.39***	−0.37***	−0.53***
(2) Outgroup individualizing		–	0.07	−0.43***	−0.34***	−0.39***
(3) Ingroup binding			–	0.27***	0.44***	0.46***
(4) Attitude bias				–	0.69***	0.72***
(5) Negative attitude bias					–	0.75***
(6) Perceived threat						–

Higher scores on political ideology reflected increased liberalism. ***p < 0.001.

#### Mediation Analyses

**FIGURE 1:**
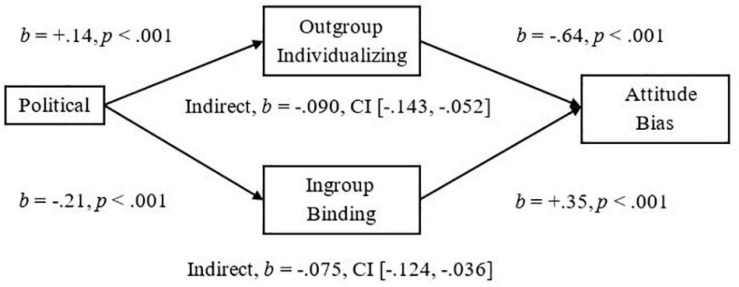
Multiple mediation of the Political Orientation to Bias relationship by the Outgroup Individualizing index and by the Ingroup Binding index using unstandardized betas.

**FIGURE 2:**
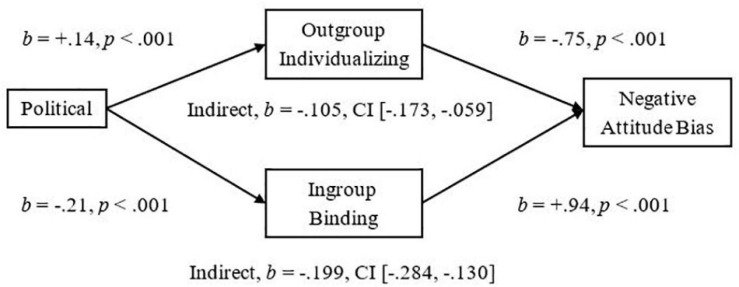
Multiple mediation of the Political Orientation to the Negative Bias relationship by the Outgroup Individualizing index and by the Ingroup Binding index using unstandardized betas.

**FIGURE 3:**
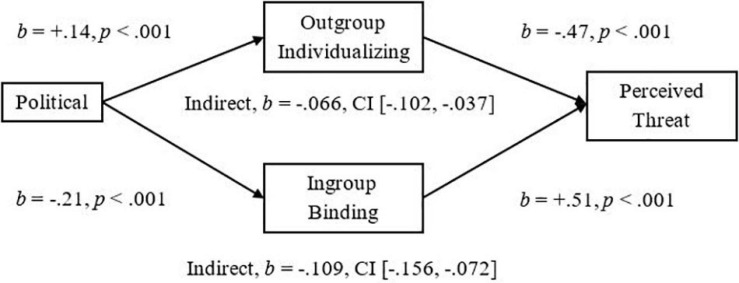
Multiple mediation of the Political Orientation to Perceived Threat relationship by the Outgroup Individualizing index and by the Ingroup Binding index using unstandardized betas.

To demonstrate the implications of this research, we examined mediators of the significant relationships we had observed between more liberalism and less Attitude Bias and between more liberalism and less Threat. We used the Outgroup-Individualizing composite of Harm and Fairness and the Ingroup-Binding composite of Ingroup-Loyalty, -Authority, and -Purity, and then tested the mediational hypotheses using unstandardized betas in the PROCESS mediation for SPSS using Bias Corrected Bootstrap analyses with 5,000 samples as suggested by Hayes (2013). As expected, we observed a significant indirect effect of Outgroup-Individualizing foundations in which more endorsement was related to less Attitude Bias, *Completely Standardized Indirect Effect (CSIE)* = −0.06, and we observed a significant indirect effect of Ingroup-Binding in which more endorsement was related to more Attitude Bias toward Pakistani-immigrants, *CSIE* = −0.11 (see Figure 1; Hypotheses 6a and b). We observed a similar pattern of indirect effects for the measure of Negative Attitude Bias, *CSIE* = −0.19 and −0.10, respectively, for Binding and Individualizing (see Figure 2). For the more liberalism to less Threat relationship, we observed the expected significant indirect effect of Outgroup-Individualizing in which more endorsement was related to less Threat, *CSIE* = −0.10, and the expected significant indirect effect of Ingroup-Binding in which more endorsement of Binding foundations was related to more Threat, *CSIE* = −0.16 (see Figure 3; Hypotheses 6c and d). All mediations were also significant when mediators were entered into separate models (see Supplementary section “Separate Outgroup-Individualizing and Ingroup-Binding Single Mediations”). See also Supplementary Section “Individualizing-Ingroup Preference Index and Binding-Ingroup Preference Index Multiple Mediations” for the predicted mediations using the manipulated Individualizing-Ingroup Preference and Binding-Ingroup Preference scores and for a discussion of these predictions.

### Discussion

The current study supports the main findings of Study 1 and showed that the ingroup- and outgroup-framing manipulation changed endorsement of the moral foundations; more liberalism was related to less Individualizing-Ingroup Preference and less Binding-Ingroup Preference. Moreover, for the separate ingroup and outgroup foundations, more liberalism predicted less endorsement of Ingroup-Binding foundations, but not more endorsement of Ingroup-Individualizing foundations. We also observed that more liberalism predicted more endorsement of Outgroup-Individualizing foundations as expected and we replicated the effect of more conservatism predicting more endorsement of Outgroup-Binding foundations, though this was a very small effect (*R*^2^ = 0.016). Overall, we observed the same general pattern on the Ingroup Preference Scores for Studies 1 and 2, even though Study 1 used abstract-ingroups and abstract-outgroups and Study 2 used a specific-outgroup and a specific-ingroup, though one that was an ingroup for all participants. This finding rules out the explanation that liberals and conservatives were thinking of very different groups and the variety of comparisons increases our confidence in the importance of the group-level in moral foundations.

The observed effects had important implications for understanding the relationship between Political Ideology and Attitudes toward and Threat from Pakistani-immigrants. For both Attitude Bias and Negative Attitude Bias, Outgroup-Individualizing foundations mediated the relationship between a more liberal ideology and less Bias toward immigrants; this supports the sparse past research that had shown that more endorsement of Individualizing foundations was related to less hostility to extreme outgroups (Baldner and Pierro, 2019). Ingroup-Binding foundations also significantly mediated the relationships between Ideology and Bias, and Ideology and Negative Bias toward immigrants, suggesting that the Ingroup-Binding foundations may also be important to consider in future research involving intergroup perceptions of immigrants. For Perceived Threat from immigrants, endorsement of Outgroup-Individualizing foundations was related to less Threat and it mediated the relationship of more liberalism predicting less Threat. Endorsement of Ingroup-Binding foundations also mediated this relationship and was related to more Threat; these effects were larger than the individualizing effects, and thus, may be particularly important for improving dialog between liberals and conservatives in relation to the topic of immigration, and may be more important than focusing on fairness judgments and harm reduction. Finally, the manipulated Individualizing-Ingroup Preference and Binding-Ingroup Preference Scores significantly predicted Attitude Bias, Negative Bias, and Threat, and each significantly mediated the observed Political to Attitudes and to Threat relationships (see Supplementary sections “Individualizing-Ingroup Preference to Attitude Bias, to Negative Bias, and to Threat Regressions, and Binding-Ingroup Preference to Attitude Bias, to Negative Bias, and to Threat regressions” and “Individualizing-Ingroup Preference Index and Binding-Ingroup Preference Index Multiple Mediations”). Overall, the first two studies provide strong and consistent evidence that liberals and conservatives are influenced differently by ingroups and outgroups, and that these differences have important implications for intergroup relations.

## Study 3

In Study 3, we wanted to replicate the significant relationships between political ideology and group-framed moral foundations, as well as the mediational effects of Ingroup-Binding foundations and Outgroup-Individualizing foundations on the political ideology to negative attitudes and political ideology to perceived threat relationships. In particular, we were interested in replicating the significant Political Ideology to Attitude Bias, to Negative Bias, to Threat effects, and the Outgroup-Individualizing and Ingroup-Binding mediations of Political Ideology to Attitude Bias, to Negative Bias, and to Perceived Threat. To further extend the current research, we added a measure of implicit bias.

### Method

#### Participants and Design

We recruited participants from the Prolific.co online platform. Based upon the previous studies, we recruited 451 participants to obtain a final sample close to 350 participants and to observe 0.8 to 0.85 power for small to medium effects, *f*^2^ = 0.03 to 0.05. As was done in Study 2, we randomly assigned participants to counterbalanced conditions for the order of ingroup- and outgroup-MFQs and for the main dependent measures using the same procedure as in Study 2. Three hundred and ninety-seven participants remained in the sample after filtering out six participants who were of Pakistani ethnicity, seven people not born in the United Kingdom, and 41 who showed inattention on the ingroup-MFQ and outgroup-MFQ as was done in Study 2 (see Graham et al., 2009). The sample had an age range of 18–74 years (*M* = 37.13, *SD* = 1.47); 54.1% were Liberal, 24.2% were moderate, and 21.7% were Conservative while 92.2% were White and 72% were Female.

#### Materials and Procedure

##### Group-framed moral foundations questionnaire (MFQ) and filler task

Participants were randomly assigned to one of eight conditions that counterbalanced the order of the ingroup- and outgroup-MFQs as was done in Studies 1 and 2, and the dependent measures (see Supplementary Table 1 sections “Distribution of Removals” and “Eight Orders to Which Participants Were Randomly Assigned in Study 3”). Participants completed either the British-Ingroup MFQ first or the Pakistani-immigrant Outgroup MFQ first; each participant then completed the filler task from Study 2 and then the version of the MFQ they had not yet completed.

##### Filler items

Participants then completed four filler questions from the need for cognition scale (Cacioppo and Petty, 1982) that had been selected because they were not significantly related to political ideology. Participants next completed either the Threat and Bias measures followed by the Implicit Bias measure, or they completed the Implicit Bias measure first followed by Threat and Bias measures (counterbalanced); after the first measure in each condition, participants completed an additional four items from the need for cognition measure as a filler.

##### Attitude bias, negative bias, and threat perceptions

The same measure of Attitude Bias toward Pakistani immigrants from Study 2 was again used in Study 3 (α = 0.93) and was followed by the same measure of Negative Bias (α = 0.96). The measure of Threat Perceptions from Pakistani immigrants was the same as in Study 2 (α = 0.94).

##### Implicit bias

Participants completed an online version of the Affective Misattribution Procedure (AMP) as a measure of indirect or implicit bias toward immigrants (Payne et al., 2005, 2008, 2010; Imhoff and Banse, 2009; Payne and Lundberg, 2014); the perspective taking measure was dropped due to time constraints produced by adding the AMP measure. In the AMP, participants saw a photograph of an Immigrant face (Pakistani/Indian face), a non-Immigrant face (White), or a neutral gray square, and the photo was quickly replaced by a Korean pictograph of a non-word letter string, letter string; the prime faces were matched for attractiveness. Similar to previous research with the AMP online (Payne et al., 2010), on each of the 72 trials, participants saw a gray dot for 500 ms to denote the beginning of a trial followed by the prime (face or gray square) for 75 ms, then the pictograph for 225 ms. A black-and-white pattern mask then appeared until participants responded with either pleasant or unpleasant as a response. Participants were instructed to ignore the faces labeled as immigrants or non-immigrants and to only judge whether or not they believed the pictograph to be more or less pleasant than average by pressing either the pleasant or unpleasant key. The 72 pictographs were presented once and the 12 immigrant faces, 12 white faces, and 12 gray squares were presented twice each (i.e., once in each block of 36 trials) and randomly paired with pictographs throughout the 72 trials.

##### Demographics and political ideology

After the outcome measures, participants answered the same demographics items from Study 2, but without the English as a second language items and with the addition of the left-right political ideology question from Study 1. They were then debriefed.

### Results

#### Ingroup Preference

We conducted separate, linear regressions using Political Ideology as the predictor and the Individualizing-Ingroup Preference and Binding-Ingroup Preference Scores as separate outcome measures. We replicated Studies 1 and 2, and found that more liberalism was related to less Individualizing-Ingroup Preference and less Binding-Ingroup Preference, which demonstrated the effectiveness of the manipulation (see Table 7).

**TABLE 7:** Standardized regression coefficients (β) for separate regression equations with political ideology predicting each framed moral foundation ingroup-preference score separately.

	**Political ideology**					
	**Separate linear regressions**				**Bootstrapping (BCa)**	
	**β**	***p*-value**	***R*^2^**	**df**	***b***	**95% CI for *b***

Individualizing-ingroup preference	−0.25***	<0.001	0.06	395	−0.12	[−0.163, −0.067]
Binding-ingroup preference	−0.38***	<0.001	0.14	395	−0.18	[−0.223, −0.133]

Higher scores on ideology reflected increased liberalism (vs. conservatism). Higher Ingroup Preference Scores indicated more investment in the moral foundation when it was framed about the ingroup. A negative regression coefficient between Preference Score and Political Ideology indicated that conservatives showed more endorsement of the moral foundation when it was framed about the ingroup as opposed to the outgroup. ***p < 0.001.

#### Separate Ingroup- and Outgroup-Framed Foundations

We replicated the negative and significant relationship between Political Ideology and Ingroup-Binding in which more liberalism was related to less endorsement of Ingroup-Binding foundations (see Table 8). While the Ingroup-Individualizing effect was now significant, it was extremely small (*R*^2^ = 0.005). We also replicated the positive and significant relationship between Political Ideology and the Outgroup-Individualizing composite in which more liberalism was related to more endorsement of Outgroup-Individualizing; we did not replicate the Outgroup-Binding effect observed in Study 1 (*R*^2^ = 0.06) or the small effect (*R*^2^ = 0.016) in Study 2 (see Supplementary Materials section “Graphs of the Individualizing and Binding Foundations for Ingroups and Outgroups” for the graphs).

**TABLE 8:** Four linear regressions with political ideology as the predictor and each Ingroup and Outgroup moral foundation entered as a separate outcome measure.

	**Political ideology**					
	**Separate linear regressions**				**Bootstrapping (BCa)**	
	**β**	***p*-value**	***R*^2^**	**df**	***b***	**95% CI for *b***

**Ingroup referent**						
Ingroup-individualizing	0.11*	=0.034	0.01	395	0.05	[0.002, 0.100]
Ingroup-binding	−0.38***	<0.001	0.14	395	−0.20	[−0.245, −0.148]
**Outgroup referent**						
Outgroup-individualizing	0.31***	<0.001	0.10	395	0.17	[0.108, 0.220]
Outgroup-binding	−0.04	=0.442	0.00	395	−0.02	[−0.065, 0.030]

*MFQ variables were re-scored so that they matched the 0–5 coding for the Moral Foundations Questionnaire*. *p < 0.05, ***p < 0.001.

#### Ideology to Bias and Threat

As predicted, we observed that more liberalism was significantly related to less Bias, *R*^2^ = 0.17, β = −0.41, *t*(395) = −8.96, *p* < 0.001, to less Negative Bias *R*^2^ = 0.12, β = −0.35, *t*(395) = −7.31, *p* < 0.001, and to less Perceived Threat, *R*^2^ = 0.25, β = −0.50, *t*(395) = −11.48, *p* < 0.001. For the AMP implicit bias analyses, we followed the standard procedure and removed an additional 14 participants who, contrary to instructions, responded using the same response key on all critical trials (Payne and Lundberg, 2014). There were 383 participants remaining for those analyses. We observed that more liberalism was significantly related to less Implicit Bias, *R*^2^ = 0.08, β = −0.28, *t*(381) = −5.75, *p* < 0.001. As predicted, we also observed that more Individualizing-Ingroup Preference and more Binding-Ingroup Preference were significantly related to more Attitude Bias, more Negative Attitude Bias, and more Threat (see Supplementary section “Individualizing-Ingroup Preference to Attitude Bias, to Negative Bias, and to Threat Regressions, and Binding-Ingroup Preference to Attitude Bias, to Negative Bias, and to Threat regressions” for the regressions and discussion of these predictions). We again included a correlation table of the main measures to provide an overall picture of their relationships (see Table 9).

**TABLE 9:** Pearson correlations (*r*) between main study variables.

	**1**	**2**	**3**	**4**	**5**	**6**	**7**
(1) Political ideology	–	0.31***	−0.38***	−0.41***	−0.35***	−0.50***	−0.28***
(2) Outgroup individualizing		–	0.08	−0.51***	−0.39***	−0.43***	−0.24***
(3) Ingroup binding			–	0.27***	0.40***	0.50***	0.25***
(4) Attitude bias				–	0.71***	0.75***	0.36***
(5) Negative attitude bias					−	0.76***	0.42***
(6) Perceived threat						–	0.41***
(7) Implicit bias							–

*Higher scores on political ideology reflected increased liberalism.* ***p < 0.001.

#### Mediation Analyses

**FIGURE 4:**
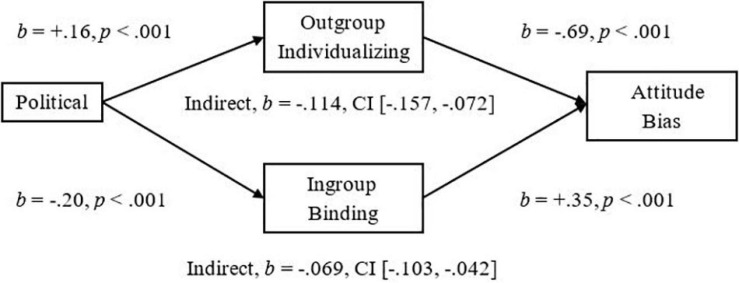
Multiple mediation of the Political Orientation to Attitude Bias relationship by the Outgroup Individualizing index and by the Ingroup Binding index using unstandardized betas.

**FIGURE 5:**
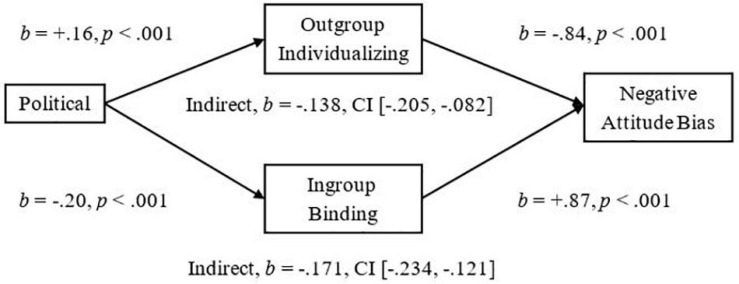
Multiple mediation of the Political Orientation to Negative Bias relationship by the Outgroup Individualizing index and by the Ingroup Binding index using unstandardized betas.

**FIGURE 6:**
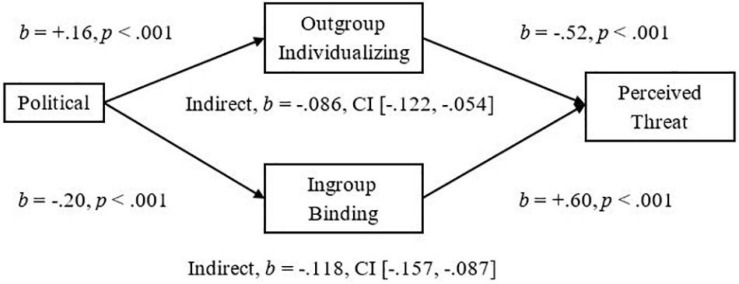
Multiple mediation of the Political Orientation to Perceived Threat relationship by the Outgroup Individualizing index and by the Ingroup Binding index using unstandardized betas.

**FIGURE 7:**
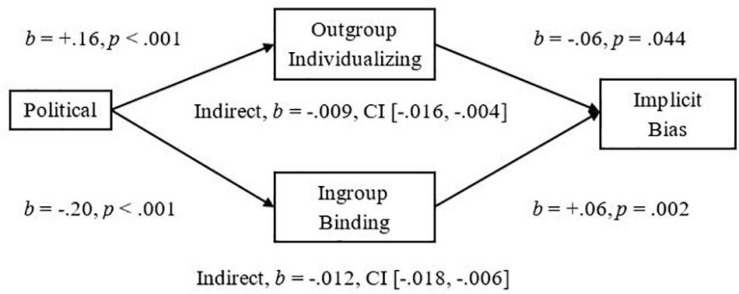
Multiple mediation of the Political Orientation to Implicit Bias relationship by the Outgroup Individualizing index and by the Ingroup Binding index using unstandardized betas.

For the Political Ideology to Attitude Bias relationship we observed the expected significant indirect effect of Outgroup-Individualizing (Outgroup-Harm and Outgroup-Fairness composite) in which more endorsement was related to less Attitude Bias toward Pakistani-immigrants, *Completely Standardized Indirect Effect (CSIE)* = −0.15 (see Figure 4). The Study 2 indirect effect of the Ingroup-Binding composite (Ingroup-Loyalty, Ingroup-Authority, and Ingroup-Purity) on Attitude Bias was replicated in Study 3 in which more endorsement was related to more Bias, *CSIE* = −0.09 (see Figure 4). Analyses on Negative Bias also yielded significant indirect effects in which more endorsement of Outgroup-Individualizing foundations was related to less Negative Bias, *CSIE* = −0.13, and more endorsement of Ingroup-Binding foundations was related to more Negative Bias, *CSIE* = −0.15 (see Figure 5). For the Political Ideology to Threat relationship, we observed the expected significant indirect effect of Ingroup-Binding in which more endorsement of Binding was related to more Threat, *CSIE* = −0.17, and we further replicated the significant indirect effect of Outgroup-Individualizing in which more endorsement was related to less Threat, *CSIE* = −0.13 (see Figure 6). For Implicit Bias (*N* = 383), we observed a significant indirect effect of both Outgroup-Individualizing foundations, *CSIE* = −0.07, and Ingroup-Binding Foundations on levels of Implicit Bias, *CSIE* = −0.09 (see Figure 7). See also Supplementary Section “Individualizing-Ingroup Preference Index and Binding-Ingroup Preference Index Multiple Mediations” for the predicted significant mediations using the manipulated Individualizing-Ingroup Preference and Binding-Ingroup Preference scores.

Finally, in all linear regressions for the moral foundations and for all the outcome variables and mediations, all patterns of data and all significant effects remained significant when using the left-right political ideology item (Studies 1 and 3). Additionally, in all studies, all the main analyses remained significant with the exception of Outgroup-Binding (Study 2), and Ingroup-Individualizing (Study 3) when correcting for 4 multiple comparisons (*p* = 0.012).

### Discussion

Study 3 replicated the pattern of results observed in Study 2 in which more liberalism was related to less Individualizing-Ingroup Preference and Binding-Ingroup Preference. Moreover, we replicated the results showing that more liberalism was related to significantly more endorsement of Outgroup-Individualizing foundations (*R*^2^ = 0.10) and less endorsement of Ingroup-Binding foundations (*R*^2^ = 0.14); these were again moderately large. Together, these results support the notion that liberals and conservatives are influenced differently by groups when making moral judgments.

Our findings suggest that there is a difference between liberals and conservatives when they consider ingroups and outgroups, especially outgroups that are low in status; liberals show more endorsement than conservatives in Individualizing-Foundations when the manipulation framed them about immigrant outgroups. In contrast, conservatives show more endorsement than liberals for Binding-Foundations when the foundations are framed about the Ingroup (for either abstract-groups in Study 1 or a British-ingroup in Studies 2 and 3). Study 3 also replicated the finding that these differing influences of ingroups and outgroups for liberals and conservatives have a meaningful influence on intergroup perceptions of immigrants. We replicated the relationship of more liberalism predicting less Attitude Bias, Negative Bias, and less Perceived Threat from immigrants. Importantly, we replicated the mediational analyses in which both the Outgroup-Individualizing and Ingroup-Binding indexes significantly mediated the effects on Attitude Bias, Negative Bias, Implicit Bias, and Perceived Threat, both together and separately as mediators. Finally, we replicated the separate regressions of the Individualizing-Ingroup Preference and the Binding-Ingroup Preference index predicting more Bias, Negative Bias, Perceive Threat, and more Implicit Bias, and that both Ingroup Preferences mediated the Political Ideology to Bias, Negative Bias, and Implicit Bias relationships (see Supplementary sections “Individualizing-Ingroup Preference to Attitude Bias, to Negative Bias, and to Threat Regressions, and Binding-Ingroup Preference to Attitude Bias, to Negative Bias, and to Threat regressions” and “Individualizing-Ingroup Preference Index and Binding-Ingroup Preference Index Multiple Mediations”).

## General Discussion

Our research was the first to demonstrate that liberals endorsed harm and fairness significantly more than conservatives when they were framed about abstract-outgroups (Study 1) or specific-outgroups (a general British ingroup and Pakistani-immigrant outgroup within the United Kingdom; Studies 2 and 3). It also demonstrates that this effect is not dependent upon only groups important to each ideology or to only high status groups (Voelkel and Brandt, 2019). The framed Ingroup-Preference Scores, which demonstrated the influence of the framing manipulation on foundation endorsement, were significant for the Individualizing-Ingroup Preference (i.e., Ingroup minus Outgroup-Individualizing scores) in each of the three studies. Moreover, when comparing Stouffer’s *z*, based upon combined *p*-values and sample sizes for all 3 studies, Individualizing-Ingroup Preference was significant (*p* = 0.0000002); liberals demonstrated significantly less and conservatives significantly more endorsement of Harm and Fairness when framed about an abstract-ingroup or a specific-British ingroup compared to an abstract-outgroup or a specific-immigrant outgroup. The separate Ingroup- and Outgroup-MFQ analyses clarified these effects; conservatives demonstrated less endorsement of Harm and Fairness foundations when framed about the outgroup, but there were few differences when framed about the ingroup. Moreover, for all 3 studies, the Outgroup-Individualizing composite was significant (Stouffer’s *z*, *p* = 0.0000004), whereas the Ingroup-Individualizing composite combined for all 3 studies was not significant (Stouffer’s *z*, *p* = 0.055) and was extremely small (aggregate *R*^2^ = 0.006). Together these findings suggest that liberals endorse Harm-reduction and Fairness values more than conservatives for abstract- and specific-outgroups, but not ingroups, and this helps to explain differences in attitudes toward immigrants and low-status groups. These findings support research indicating that liberals support social justice concerns across a range of groups (Janoff-Bulman and Carnes, 2016).

Our research was the first to demonstrate that conservatives showed significantly more endorsement of Loyalty, Authority, and Purity when they were framed about abstract-ingroups (Study 1) and about a specific-British ingroup (Studies 2 and 3). Across all three studies, liberals showed less endorsement and conservatives more endorsement of Loyalty, Authority, and Purity regardless of whether the ingroups were abstract or specific. Overall, Binding-Ingroup Preference (i.e., Ingroup minus Outgroup-Binding scores) was significant for each of the three studies, which once again demonstrated the effectiveness of the framing manipulation; Stouffer’s *z*, based upon *p*-values and sample size combined for all three studies, showed that Binding-Ingroup Preference was significant (*p* < 0.0000001). Once again, the separate ingroup- and outgroup-foundation analyses provided insight here. The average Ingroup-Binding effects were large and robust. For all three studies, Ingroup-Binding was significant (*p* = 0.0000002) and large (aggregate *R*^2^ = 0.198), whereas the Outgroup-Binding effect was significant (*p* = 0.02), but very small (aggregate *R*^2^ = 0.017). Furthermore, the Outgroup-Binding effect for the two, specific-group studies was non-significant and extremely small (aggregate *R*^2^ = 0.008, Stouffer’s *z*, *p* = 0.101). Overall, liberals and conservatives differ on using ingroups and outgroups depending upon the foundations considered and these differences have important consequences for immigrant intergroup contexts and cultural divides.

In our studies, a more liberal political ideology was related to less Attitude Bias, less Negative Bias, and less Perceived Threat from immigrants (Studies 2 and 3), and to less Implicit Bias toward immigrants (Study 3); Stouffer’s *z* analyses showed that all effects were strong for the combination of Studies 2 and 3 (all *p*s < 0.000007). We were the first to demonstrate that the relationships between Political Ideology and Attitude Bias, Negative Bias, and Implicit Bias toward immigrants, as well as Perceived Threat from immigrants were mediated by more endorsement of Outgroup Individualizing foundations and British-Ingroup Binding foundations, both together and in separate mediations (Studies 2 and 3). They were also mediated by Individualizing-Ingroup Preference and Binding-Ingroup Preference composites (Studies 2 and 3). Importantly, we demonstrated that Attitude Bias, Negative Bias, Threat, and Implicit Bias toward immigrants were predicted by both Individualizing- and Binding-Ingroup Preference composites (all *p*s < 0.0000073 for Stouffer’s *z*). Finally, all linear regression and mediational analyses were replicated for all 3 studies when those who failed the MFQ attention items were not removed from the samples, with the exception of the Binding-Ingroup Preference composite in Study 3. This effect became non-significant for Attitude Bias and Negative Bias in the multiple mediations, but was significant in the single mediations. Overall the findings have important implications for interventions designed to improve dialogs in relation to immigrants.

In our research, we demonstrated that the types of groups that come to mind matter for all five foundations within the moral foundations questionnaire. Liberals showed more endorsement than conservatives in Harm reduction and Fairness when both abstract-outgroups and an immigrant-outgroup were the focus of moral judgments, while conservatives showed more endorsement in Loyalty, Authority, and Purity when ingroups were the focus (both for abstract-ingroups and for a more neutral British-ingroup). The observed differences for harm and fairness are in line with other research showing that liberals show more promotion focus when searching novel stimuli, show higher levels of openness to experience, and show more endorsement of social equality and more acceptance of change. These effects are particularly true for low-status groups such as immigrants, but could also be true for mid-to-high status outgroups, though this idea would need to be tested in future research (Duckitt, 2001; Jost et al., 2003, 2017; Thórisdóttir et al., 2007; Duckitt and Sibley, 2009; Federico et al., 2009; Shook and Fazio, 2009; Sibley et al., 2012; Portes, 2015; Baldner and Pierro, 2019). The observed differences for loyalty, authority, and purity are also in accordance with research showing that conservatives may attend to and may be more vigilant for threat or danger cues that may impact on loyalty, authority, or purity concerns relating to ingroup boundaries (Jost et al., 2003, 2007; Duckitt and Sibley, 2009; Van Leeuwen and Park, 2009; Hibbing et al., 2014). In Studies 2 and 3, we observed that more Perceived Threat from immigrants (i.e., combination of perceived symbolic threat and realistic threat) was associated with a more conservative ideology. This perception of threat may reflect wanting to protect group boundaries, customs, and traditions, and to minimize exposing one’s group to risk. In a democracy, this risk minimization will need to be balanced with acceptance of risk. This is especially true in societies with birth rates below population replacement that will continue to rely upon immigration to maintain population growth, which in addition to increased efficiency, is often closely tied to economic growth.

In considering the consequences of these tendencies, we demonstrated that liberals showed less Attitude Bias, both Explicit and Implicit, and less Negative Bias toward immigrants. Moreover, liberals’ higher endorsement of Harm reduction and Fairness should be associated with more willingness to include low-status others in their harm and fairness judgments. These endorsements should also be associated with less bias toward low-status outgroups, and outgroups in general, though there may be some exceptions for higher status outgroups (Brandt et al., 2014; Voelkel and Brandt, 2019). Future research will need to further investigate the status range of outgroups that might be included in these judgments. In Studies 2 and 3, we were the first to demonstrate that the Political Ideology relationship to Attitude Bias, both Explicit and Implicit, was mediated by endorsement of Outgroup-Individualizing foundations of harm and fairness related to immigrants. Moreover, Outgroup-Individualizing foundations mediated the relationship between Political Orientation and Negative Bias. Thus, more inclusion of immigrant outgroups in moral judgments of harm and fairness is important for reducing both attitude bias and negative attitudes. Ingroup-Binding foundations also mediated the relationships between Political Ideology and Implicit, and Explicit Bias, and Negative Bias.

We included a measure of explicit bias toward immigrants within the studies in order to compare across studies and to accommodate a broader, online sample. The use of the online context allowed for a larger and more diverse sample than our lab context allowed, but in Study 2, we did not have the ability to use the implicit measure. Overall, we felt that the continued inclusion of the explicit measure was warranted for a number of reasons. First, we did not observe floor effects in which all participants responded at the bottom of the scale. In fact, we observed substantial effect sizes for these measures (*R*^2^ = 0.15 and *R*^2^ = 0.17 in Studies 2 and 3 for explicit bias, *R*^2^ = 0.14, and *R*^2^ = 0.12 for negative bias in Studies 2 and 3). Second, we wanted to keep a few measures the same between studies in order to compare across studies. Third, our results were in line with other research on political ideology and attitudes in general and in relation to low-status groups, and we replicated our effects with an implicit measure toward immigrants (Cunningham et al., 2004; Nisbet and Shanahan, 2004; Luguri et al., 2012; Webster et al., 2014). However, it is possible that liberals may have been reporting less bias than they actually felt, though we believe this was not the case because they also showed little bias on the implicit measure (just below 0% bias on the AMP compared to above 10% for conservatives in Study 3). It is also possible that conservatives’ reporting of bias was deliberately higher on the explicit measures, which increased the overall effect size; they may have felt okay about expressing their attitudes against this group in the modern climate (Brexit or the current social climate in the United States and Europe).

In our studies we used both abstract- and specific-group framing of the target people in the moral foundations questionnaire in order to establish the effects of framing on endorsement of moral foundations by liberals and conservatives. We found differences in which liberals endorsed outgroup-individualizing foundations more while conservatives endorsed ingroup-binding foundations more. Removing all ambiguity in group-level within the MFQ, however, remains a challenge. Some of our abstract MFQ items may have retained some level of ambiguity. For example, an item assessing ingroup authority asked “Whether or not someone showed a lack of respect for authority of my ingroup” with the equivalent outgroup authority item asking “Whether or not someone showed a lack of respect for authority of an outgroup.” Thus, there still exists a level of ambiguity because some participants may interpret the ‘someone’ as being an ingroup or outgroup member. While this may add some variance to responses, the target group (ingroup or outgroup) was clearly distinguished within the questionnaire and those groups were the focus of the manipulation. Importantly, we did observe theoretically predicted differences due to the ingroup- versus outgroup-framing, and we did observe similar patterns in endorsement for the abstract-group framing (Study 1) and the specific-group framing (Studies 2 and 3). We, therefore, believe that our results demonstrate true and consistent differences even with the presence of additional variation. Moreover, in Studies 2 and 3, the ingroup–outgroup manipulations related to intergroup attitudes and bias as predicted by Intergroup Threat Theory (Stephan et al., 2015). Nonetheless, there is still potential to reduce ambiguities in the group framing further in future research to assess whether it would change these interpretations.

The differences in relying on ingroups and outgroups when thinking about morality can be helpful in understanding differences in reactions to immigrants and other outgroups, such as ethnic outgroups, which we suspect may show similar patterns to those for immigrants, especially for low-status groups and for people who view prototypical members of their society as very homogenous. Our research can help inform how to frame discussions on this topic, and other topics related to ethnicity and intergroup relations, and a number of other issues dividing liberals and conservatives. For attitudes toward immigrants, significant outgroup-individualizing and ingroup-binding mediational analyses provide insight here. Highlighting the United Kingdom’s history or any country’s history of the inclusion of others within society and emphasizing reduction of harm and increased fairness could help to reduce this attitude effect. However, this may work with only roughly half the population (moderates to liberal) who do not focus as much on ingroup-binding values, so other ways of discussing these issues will also need to be sought to improve public dialog. For Implicit attitudes, discussions could be potentially framed with either an Outgroup-Individualizing focus or a Binding focus. However, given that perceived threat was strongly linked to political ideology and to the binding foundations and that binding mediated the political ideology to threat relationship, reducing threat may be one of the most important focuses for future research and future dialogs.

When discussing topics related to immigrants, we should be cognizant to strike a balance between threat acceptance and threat minimization to begin to bridge some of the partisan divides. Our research, particularly in relation to the ingroup-binding mediations and threat, highlights the notion that differences in threat perceptions may relate to different levels of comfort in risk acceptance and risk minimization between liberals and conservatives. While neither one of these preferences is necessarily better than the other, there are important consequences in a world that is becoming more socially and culturally diverse. Such a focus could provide a common avenue for discussing partisan differences in a constructive manner, and in such a way that balances risk acceptance and risk minimization. One such approach that may be effective, especially among conservatives, is reducing threats to binding motivations and reducing symbolic and realistic threats, especially toward ingroups. To offset perceived realistic threats, acknowledging the minimization of undue risks from the outset may allow for the positive economic impacts of immigrants and immigration, and the avoidance of negative impacts that may exist is some places (e.g., the increased need for English as second language instruction). To offset symbolic threat to ingroups, discussions can be focused upon the ideas that efforts would be taken to ensure law, order, and fairness, and to help immigrants learn the country-specific systems in order to contribute to the country’s prosperity and shared governing principles. For liberals, either the inclusion of others framing or the threat reduction framing should help to reduce bias because liberals can also show increased bias when threats are explicitly highlighted (Van de Vyver et al., 2016). Of course, within open and democratic societies, we would need to balance the minimization of too much risk with the risk of including others. This will continue to be a challenge as immigration will likely continue and ethnic, cultural, and linguistic diversity will likely increase. In these contexts, finding ways to have constructive dialogs will be important to moving the debates forward, and should provide avenues for tackling other intergroup issues as well as other general topics that divide liberals and conservatives.

## Conclusion

Our research adds to recent research showing differences between liberals and conservatives as being influenced by social context (Janoff-Bulman and Carnes, 2013, 2016; Frimer et al., 2014; Morgan et al., 2014; Talaifar and Swann, 2019). Importantly, our research highlights the significance of using outgroups and general ingroups differently when making moral judgments and the impact that it may have on immigrants and intergroup situations. Based upon group and moral judgments, we have identified avenues to pursue that may improve intergroup dialogs on these issues. Future work will need to investigate this idea more thoroughly to ask how and when our moral decisions tend to be tied to our group loyalties, and what influence that has for intergroup relations and social perceptions in general.

## Data Availability Statement

The original datasets generated for this study are included in the article/Supplementary Material, further inquiries can be directed to the corresponding author.

## Ethics Statement

The studies involving human participants were reviewed and approved by the studies were conducted in accordance with the recommendations of the BPS guidelines and the Science, Technology, Engineering and Mathematics Ethical Review Committee, with written informed consent from all participants. All participants gave written informed consent in accordance with the Declaration of Helsinki. The protocol was approved by the STEM Ethical Review Committee at the University of Birmingham. The patients/participants provided their written informed consent to participate in this study.

## Author Contributions

BS conceived of the original study ideas. DM provided significant input into the development of the research. Study design and analysis of data was conducted by DM with assistance by BS. Overall, BS and DM were equally involved in writing up the studies for submission. Both authors contributed to the article and approved the submitted version.

## Conflict of Interest

The authors declare that the research was conducted in the absence of any commercial or financial relationships that could be construed as a potential conflict of interest.

## Footnotes

1 Only 5 of 30 items of the current MFQ reference one’s group, which allows for significant interpretation of the target group in each question (Graham et al., 2008; retrieved after July 2008).

2 The pattern of results for the regression analyses was similar for the 153-participant sample and the full 162 participant sample, and all significant results remained significant.

3 As part of a separate exploratory study, participants completed the Behavioral Activation Scale (BAS), Model of Moral Motives (MMM), Affect Misattribution Procedure (AMP), Belief in a Dangerous World scale (BDW), and some questions about the purpose of the study, whether tasks were related, if they’d been in a similar study, how many studies they had been in today, how they were feeling today, and contact with a minority group. In Study 1, we replicated the AMP analyses of Study 3 for Political to AMP (*R*^2^ = 0.04).
