# Supplementary material for: Moving Morality Beyond the In-Group: Liberals and Conservatives Show Differences on Group-Framed Moral Foundations and These Differences Mediate the Relationships to Perceived Bias and Threat
Source: Front Psychol. 2021 Apr 21;12:579908. doi: 10.3389/fpsyg.2021.579908 (PMC8096906; doi:10.3389/fpsyg.2021.579908)
Supplement: Supplementary file 7 [file Table_1.DOCX]

**Table1 Supplemental Materials and Analyses for Stewart & Morris (2020)**

**Section 1: Distribution of removals.**

**Study 1**: Five participants were removed who were in the Ingroup-First condition and four participants were removed who were in the Outgroup-First within participants condition.

**Study 2**: Four Pakistani participants were removed (1 from the Ingroup-First condition and 3 from the Outgroup-First within participants condition). Three participants who were not born in the UK were removed (1 from the Ingroup-First condition and 2 from the Outgroup-First within participants condition). Thirty-seven participants who failed the MFQ attention checks were removed (21 from the Ingroup-First condition and 16 from the Outgroup-First within participants condition).

**Study 3**: Six Pakistani participants were removed (4 from the Ingroup-First condition and 2 from the Outgroup-First within participants condition). Seven participants who were not born in the UK were removed (2 from the Ingroup-First condition and 5 from the Outgroup-First within participants condition). Thirty-seven participants who failed the MFQ attention checks were removed (19 from the Ingroup-First condition and 21 from the Outgroup-First within participants condition).

**Section 2:**

**Study 1: The (Abstract) Ingroup and Outgroup Framed MFQs**

Note: Measure adapted based on Graham, Haidt and Nosek, (2008). For data analysis, the presentation software coded this scale from 1 = Not at all relevant, and 6 = Extremely relevant, and the judgment scale was coded 1 = Strongly disagree, and 6 = Strongly agree). The Ingroup- and Outgroup-MFQs differ only in the target being Ingroup or Outgroup.

**Ingroup MFQ**

Part 1. When you decide whether something is right or wrong, to what extent are the following considerations relevant to your thinking? Please rate each statement using this scale:

[0] = not at all relevant (i.e., has nothing to do with my judgments of right & wrong)

[1] = not very relevant

[2] = slightly relevant

[3] = somewhat relevant

[4] = very relevant

[5] = extremely relevant (i.e., a very important factor in judging right & wrong)

______ Whether or not someone in my ingroup suffered emotionally

______ Whether or not some people from my ingroup were treated differently than others

______ Whether or not someone's action showed love for his or her country, which is also my ingroup

______ Whether or not someone showed a lack of respect for authority of my ingroup

______ Whether or not someone violated my ingroup's standards of purity and decency

______ Whether or not someone was good at maths

______ Whether or not someone cared for someone weak or vulnerable from my ingroup

______ Whether or not someone acted unfairly towards my ingroup

______ Whether or not someone did something to betray his or her ingroup

______ Whether or not someone conformed to the traditions of my ingroup's society

______ Whether or not someone did something my ingroup would consider disgusting

______ Whether or not someone was cruel to my ingroup

______ Whether or not someone from my ingroup was denied his or her rights

______ Whether or not someone showed a lack of loyalty to my ingroup

______ Whether or not an action caused chaos or disorder for my ingroup

______ Whether or not someone acted in a way that my ingroup's God would approve of

Part 2. Please read the following sentences and indicate your agreement or disagreement:

[0] [1] [2] [3] [4] [5]

Strongly Moderately Slightly Slightly Moderately Strongly

disagree disagree disagree agree agree agree

______ Compassion for those who are suffering in one's ingroup is the most crucial virtue.

______ When the government makes laws, the number one principle should be ensuring that everyone in my ingroup is treated fairly.

______ I am proud of my country's and my ingroup's history.

______ Respect for authority of one's ingroup is something all children need to learn.

______ People should not do things that my ingroup considers disgusting, even if no one is harmed

______ It is better to do good than to do bad.

______ One of the worst things a person could do is hurt a defenseless animal belonging to a member of my ingroup.

______ Justice for my ingroup is the most important requirement for a society.

______ People from my ingroup should be loyal to their family members, even when their family has done something wrong.

______ Men and women from my ingroup each have different roles to play in society.

______ I would call some acts wrong on the grounds that they are unnatural as judged by my ingroup

______ It can never be right to kill a human being from one's ingroup.

______ I think it's morally wrong that rich children from my ingroup inherit a lot of money while poor children from my ingroup inherit nothing.

______ It is more important to be a team player with one's ingroup than to express oneself.

______ If I were a soldier and disagreed with my ingroup commanding officer's orders, I would obey anyway because that is my duty.

______ Chastity within my ingroup is an important and valuable virtue.

**Outgroup MFQ**

Note: Measure adapted based on Graham, Nosek, and Haidt (2008). For data analysis, the presentation software coded this scale from 1 = Not at all relevant, and 6 = Extremely relevant, and the judgment scale was coded 1 = Strongly disagree, and 6 = Strongly agree

Part 1. When you decide whether something is right or wrong, to what extent are the following considerations relevant to your thinking? Please rate each statement using this scale:

[0] = not at all relevant (i.e., has nothing to do with my judgments of right & wrong)

[1] = not very relevant

[2] = slightly relevant

[3] = somewhat relevant

[4] = very relevant

[5] = extremely relevant (i.e., a very important factor in judging right & wrong

______ Whether or not someone in an outgroup suffered emotionally

______ Whether or not some people from an outgroup were treated differently than others

______ Whether or not someone's action showed love for his or her country, which is also an outgroup to my country

______ Whether or not someone showed a lack of respect for authority of an outgroup

______ Whether or not someone violated an outgroup's standards of purity and decency

______ Whether or not someone was good at maths

______ Whether or not someone cared for someone weak or vulnerable from an outgroup

______ Whether or not someone acted unfairly towards an outgroup

______ Whether or not someone did something to betray an outgroup

______ Whether or not someone conformed to the traditions of an outgroup's society

______ Whether or not someone did something an outgroup would consider disgusting

______ Whether or not someone was cruel to an outgroup

______ Whether or not someone from an outgroup was denied his or her rights

______ Whether or not someone showed a lack of loyalty to an outgroup

______ Whether or not an action caused chaos or disorder for an outgroup

______ Whether or not someone acted in a way that an outgroup's God would approve of

Part 2. Please read the following sentences and indicate your agreement or disagreement:

[0] [1] [2] [3] [4] [5]

Strongly Moderately Slightly Slightly Moderately Strongly

disagree disagree disagree agree agree agree

______ Compassion for those who are suffering in an outgroup is the most crucial virtue.

______ When the government makes laws, the number one principle should be ensuring that everyone in an outgroup is treated fairly.

______ I am proud of different countries’ and outgroups’ histories.

______ Respect for authority of one's outgroups is something all children need to learn.

______ People should not do things that an outgroup considers disgusting, even if no one is harmed.

______ It is better to do good than to do bad.

______ One of the worst things a person could do is hurt a defenseless animal belonging to a member of an outgroup.

______ Justice for an outgroup is the most important requirement for a society.

______ People from an outgroup should be loyal to their family members, even when their family has done something wrong.

______ Men and women from an outgroup each have different roles to play in society.

______ I would call some acts wrong on the grounds that they are unnatural as judged by an outgroup

______ It can never be right to kill a human being from an outgroup.

______ I think it's morally wrong that rich children from an outgroup inherit a lot of money while poor children from an outgroup inherit nothing.

______ It is more important to be a team player with an outgroup than to express oneself.

______ If I were a soldier and disagreed with orders from a commanding officer who belonged to an outgroup, I would obey anyway because that is my duty.

______ Chastity within an outgroup is an important and valuable virtue.

**Studies 2 and 3: The (Specific) Ingroup and Outgroup MFQs**

Note: Measure adapted based on Graham, Haidt and Nosek, (2008). For data analysis, the presentation software coded this scale from 1 = Not at all relevant, and 6 = Extremely relevant, and the judgment scale was coded 1 = Strongly disagree, and 6 = Strongly agree). The Ingroup- and Outgroup-MFQs differ only in the target being Ingroup or Outgroup.

**Specific-Ingroup MFQ**

Part 1. When you decide whether something is right or wrong, to what extent are the following considerations relevant to your thinking? Please now rate the following statements:

0 = not at all relevant (This consideration has nothing to do with my judgments of

right and wrong)

1 = not very relevant

2 = slightly relevant

3 = somewhat relevant

4 = very relevant

5 = extremely relevant (This is one of the most important factors when I judge right

and wrong)

___Whether or not someone from Britain suffered emotionally.

___ Whether or not some British people were treated differently than others.

___ Whether or not someone from Britain showed love for Britain.

___ Whether or not someone showed a lack of respect for authority of people from Britain.

___ Whether or not someone violated British standards of purity and decency.

___ Whether or not someone was good at maths

___ Whether or not someone cared for someone weak or vulnerable from Britain.

___ Whether or not someone acted unfairly towards people from Britain.

___ Whether or not someone did something to betray people from Britain.

___ Whether or not someone conformed to the traditions of British society.

___ Whether or not someone did something people from Britain would consider disgusting.

___ Whether or not someone was cruel to people from Britain.

___ Whether or not someone from Britain was denied his or her rights.

___ Whether or not someone showed a lack of loyalty to people from Britain.

___ Whether or not an action caused chaos or disorder for people from Britain.

___ Whether or not someone acted in a way that Britain's God would approve of.

Part 2. Please read the following sentences and indicate your agreement or disagreement:

0 = Strongly disagree

1 = Moderately disagree

2 = Slightly disagree

3 = Slightly agree

4 = Moderately agree

5 = Strongly agree

___ Compassion for those who are suffering in Britain is the most crucial virtue.

___ When the government makes laws, the number one principle should be ensuring that

everyone in Britain is treated fairly.

___ I am proud of Britain’s history.

___ Respect for authority of Britain is something all children need to learn.

___ People should not do things that people from Britain considers disgusting, even if no

one is harmed.

___ It is better to do good than to do bad.

___ One of the worst things a person could do is hurt a defenceless animal belonging to a

member of Britain.

___ Justice for people from Britain is the most important requirement for society.

___ People from Britain should be loyal to their family members, even when their family has

done something wrong.

___ Men and women from Britain each have different roles to play in society.

___ I would call some acts wrong on the grounds that they are unnatural as judged by

people from Britain.

___It can never be right to kill a human being from Britain.

___ I think it's morally wrong that rich children in Britain inherit a lot of money while poor

children in Britain inherit nothing.

___ It is more important to be a team player with Britain than to express oneself.

___ If I were a soldier and disagreed with my British commanding officer's orders, I would

obey anyway because that is my duty.

___ Chastity within Britain is an important and valuable virtue.

**Specific-Outgroup MFQ**

When you decide whether something is right or wrong, to what extent are the following considerations relevant to your thinking? Please now rate the following statements:

0 = not at all relevant (This consideration has nothing to do with my judgments of right and wrong)

1 = not very relevant

2 = slightly relevant

3 = somewhat relevant

4 = very relevant

5 = extremely relevant (This is one of the most important factors when I judge right and wrong)

___ Whether or not someone in the Pakistani immigrant group suffered emotionally.

___ Whether or not some people from the Pakistani immigrant group was treated

differently than others.

___ Whether or not someone from the Pakistani immigrant group showed love for his or her

country.

___ Whether or not someone showed a lack of respect for authority of the Pakistani

immigrant group.

___ Whether or not someone violated the Pakistani immigrant group's standards of purity

and decency.

___ Whether or not someone was good at maths.

___ Whether or not someone cared for someone weak or vulnerable from the Pakistani

immigrant group.

___ Whether or not someone acted unfairly towards the Pakistani immigrant group.

___ Whether or not someone did something to betray the Pakistani immigrant group.

___ Whether or not someone conformed to the traditions of the Pakistani immigrant

group's society.

___ Whether or not someone did something the Pakistani immigrant group would consider

disgusting.

___ Whether or not someone was cruel to the Pakistani immigrant group.

___ Whether or not someone from the Pakistani immigrant group was denied his or her

rights.

___ Whether or not someone showed a lack of loyalty to the Pakistani immigrant group.

___ Whether or not an action caused chaos or disorder for the Pakistani immigrant group.

___ Whether or not someone acted in a way that the Pakistani immigrant group's God

would approve of.

Part 2. Please read the following sentences and indicate your agreement or disagreement:

0 = Strongly disagree

1 = Moderately disagree

2 = Slightly disagree

3 = Slightly agree

4 = Moderately agree

5 = Strongly agree

___ Compassion for those who are suffering in the Pakistani immigrant group is the most

crucial virtue.

___ When the government makes laws, the number one principle should be ensuring that

everyone in the Pakistani immigrant group is treated fairly.

___ I am proud of the Pakistani immigrant group’s history.

___ Respect for authority of the Pakistani immigrant group is something all children need to

learn.

___ People should not do things that the Pakistani immigrant group considers disgusting,

even if no one is harmed.

___ It is better to do good than to do bad.

___ One of the worst things a person could do is hurt a defenceless animal belonging to a

member of the Pakistani immigrant group.

___ Justice for the Pakistani immigrant group is the most important requirement for society.

___ People from the Pakistani immigrant group should be loyal to their family members,

even when their family has done something wrong.

___ Men and women from the Pakistani immigrant group each have different roles to play

in society.

___ I would call some acts wrong on the grounds that they are unnatural as judged by the

Pakistani immigrant group.

___ It can never be right to kill a human being from the Pakistani immigrant group.

___ I think it's morally wrong that rich children in the Pakistani immigrant group inherit a lot

of money while poor children in the Pakistani immigrant group inherit nothing.

___ It is more important to be a team player with the Pakistani immigrant group than to

express oneself.

___ If I were a soldier and disagreed with orders from a commanding officer who belonged

to the Pakistani immigrant group, I would obey anyway because that is my duty.

___ Chastity within the Pakistani immigrant group is an important and valuable virtue.

**Section 3: Reliability Analyses**

**Study 1**:

A reliability analysis was performed on all subscales for both the ingroup- and outgroup-MFQs. For the outgroup version, the reliability for each foundation was α = .68 for Harm, α = .72 for Fairness, α = .67 for Loyalty, α = .65 for Authority, and α = .73 for Purity. For the ingroup-MFQ, reliability for each foundation was α = .60 for Harm, α = .52 for Fairness, α = .72 for Loyalty, α =.67 for Authority, and α = .80 for Purity. While it is of note that a few alpha values were slightly low, many other moral foundations researchers have also demonstrated a mixture of low to high reliabilities. Federico, Weber, Ergun, & Hunt (2013) observed a range of reliabilities from α = .52 to .73 across two separate samples. As Federico et al (2013) noted, lower reliability in the moral foundations subscales is partially due to the way the scale was created to capture broad moral dimensions instead of maximizing internal consistency. Furthermore, we observed similar reliabilities to Graham et al., 2009, in which they measured the relationship between moral foundations and political orientation (see below), and a similar range of reliabilities to other previous work (Graham et al., 2012; Hirsh, De Young, Xu, & Peterson, 2010).

Graham et al. (2009) divided the foundations into the 3 item (per foundation) relevance subscale (Study 1) and found reliabilities of α = .62 for Harm, α = .67 for Fairness, α = .59 for Loyalty, α = .39 for Authority, and α = .70 for Purity. In our study for the Outgroup relevance subscales (3 items per foundation) we found reliabilities of α = .74 for Harm, α = .78 for Fairness, α = .70 for Loyalty, α = .60 for Authority and α = .66 for Purity. For the ingroup relevance subscales we found reliability values of α = .63 for Harm, α = .60 for Fairness, α = .67 for Loyalty, α = .51 for Authority and α = .68 for Purity. Graham et al. (2009) also investigated the reliabilities for the 4 item judgment scales (Study 2) and found reliabilities of α = .50 for Harm, α = .39 for Fairness, α = .24 for Loyalty, α = .64 for Authority, and α = .74 for Purity. In our study for the Outgroup Judgement subscales (3 items per foundation) we found reliabilities of α = .51 for Harm, α = .52 for Fairness, α = .44 for Loyalty, α = .54 for Authority and α = .58 for Purity. For the Ingroup Judgment subscales we found reliabilities of α = .44 for Harm, α = .40 for Fairness, α = .49 for Loyalty, α = .52 for Authority and α = .70 for Purity.

**Study 2**:

Reliabilities for subscales of the MFQ were found to be acceptable and comparable across versions for Harm (Ingroup Harm α = .71, Outgroup Harm α = .72), Fairness (Ingroup Fairness α = .73, Outgroup Fairness α = .71), Loyalty (Ingroup Loyalty α = .78, Outgroup Loyalty α = .65), Authority (Ingroup Authority α = .74, Outgroup Authority α = .62), and Purity (Ingroup Purity α = .83, Outgroup Purity α = .80).

**Study 3**:

Reliability analyses for each subscale of the Ingroup MFQ and Outgroup MFQ were as follows Harm (Ingroup Harm α = .75, Outgroup Harm α = .75), Fairness (Ingroup Fairness α = .75, Outgroup Fairness α = .78), Loyalty (Ingroup Loyalty α = .83, Outgroup Loyalty α = .73), Authority (Ingroup Authority α = .76, Outgroup Authority α = .66), Purity (Ingroup Purity α = .83, Outgroup Purity α = .82).

**Section 4: Graphs of the Individualizing and Binding Foundations for Ingroups and Outgroups**

**Study 1**

|  | Political Ideology | | | | | |  |
| --- | --- | --- | --- | --- | --- | --- | --- |
|  | | Linear Regressions | | |  | Bootstrapping (BCa) | |
|  | | ***β*** | ***p-value*** | ***R^2^*** | ***df*** | ***b*** | ***95% CI for b*** |
| ingroup-Individualizing | | .01 | = .870 | .00 | 151 | .01 | [-.056, .060] |
|  | |  |  |  |  |  |  |
| outgroup-Individualizing | | .24** | = .003 | .06 | 151 | .10 | [.031, .174] |
|  | |  |  |  |  |  |  |
| ingroup-Binding | | -.52*** | < .001 | .27 | 151 | -.26 | [-.331, -.196] |
|  | |  |  |  |  |  |  |
| outgroup-Binding | | -.25** | = .002 | .06 | 151 | -.12 | [-.188, -.043] |

**Study 2 Graphs**

|  | Political Ideology | | | | | | | | |  |
| --- | --- | --- | --- | --- | --- | --- | --- | --- | --- | --- |
|  | | Linear Regressions | | | |  | | Bootstrapping (BCa) | | |
|  | | | ***β*** | ***p-value*** | ***R^2^*** | | ***df*** | ***b*** | ***95% CI for b*** | |
| ingroup-Individualizing | | | .08 | = .175 | .01 | | 305 | .04 | [-.019, .090] | |
|  | | |  |  |  | |  |  |  | |
| outgroup-Individualizing | | | .29*** | < .001 | .08 | | 305 | .14 | [.085, .195] | |

| ingroup-Binding | -.44*** | < .001 | .19 | 305 | -.21 | [-.265, -.160] |
| --- | --- | --- | --- | --- | --- | --- |
|  |  |  |  |  |  |  |
| outgroup-Binding | -.13* | = .029 | .02 | 305 | -.05 | [-.103, -.006] |

**Study 3 Graphs**

|  | Political Ideology | | | | | | | | | |  |
| --- | --- | --- | --- | --- | --- | --- | --- | --- | --- | --- | --- |
|  | | | Linear Regressions | | | | | Bootstrapping (BCa) | | | |
|  | | ***β*** | | ***p-value*** | ***R^2^*** | ***df*** | ***b*** | | ***95% CI for b*** | | |
| ingroup-Individualizing | | .11* | | = .034 | .01 | 395 | .05 | | | [.002, .100] | |
|  | |  | |  |  |  |  | | |  | |
| outgroup-Individualizing | | .31*** | | < .001 | .10 | 395 | .17 | | | [.108, .220] | |

| ingroup-Binding | -.38*** | < .001 | .14 | 395 | -.20 | [-.245, -.148] |
| --- | --- | --- | --- | --- | --- | --- |
|  |  |  |  |  |  |  |
| outgroup-Binding | -.04 | = .442 | .00 | 395 | -.02 | [-.065, .030] |

**Section 5: Separate Outgroup-Individualizing and Ingroup-Binding single mediations**.

**Study 2**:

Three separate Outgroup-Individualizing mediations of Political Ideology to Bias, to Negative Bias, and to Threat were conducted. We observed a significant indirect effect of the Outgroup-Individualizing foundations in which more investment was related to less Bias, *b* = -.0788, CI [-.1257, -.0446], *Completely Standardized Indirect Effect (CSIE)* = -.1002, less Negative Bias, *b* = -.0765, CI [-.1365, -.0386], *CSIE* = -.0726, and less Perceived Threat, *b* = -.0504, CI [-.0834, -.0267], *CSIE* = -.0745.

Three separate Ingroup-Binding mediations of Political Ideology to Bias, to Negative Bias, and to Threat were conducted. We observed a significant indirect effect of the Ingroup-Binding foundations in which more investment was related to more Bias, *b* = -.0423, CI [-.0919, -.0012], *CSIE* = -.0537, (Ingroup-Binding to Bias *b* = .1992), more Negative Bias, *b* = -.1601, CI [-.2437, -.0984], *CSIE* = -.1519, (Ingroup-Binding to Negative Bias *b* = .7544), and more Perceived Threat, *b* = -.0844, CI [-.1321, -.0499], *CSIE* = -.1249, (Ingroup-Binding to Threat *b* = .3979).

**Study 3**:

Four separate Outgroup-Individualizing mediations of Political Ideology to Bias, to Negative Bias, to Implicit Bias, and to Threat were conducted. We observed a significant indirect effect of the Outgroup-Individualizing foundations in which more investment was related to less Bias, *b* = -.1008, CI [-.1423, -.0633], *Completely Standardized Indirect Effect (CSIE)* = -.1338, less Negative Bias, *b* = -.1069, CI [-.1717, -.0576], *CSIE* = -.0970, less Implicit Bias, *b* = -.0068, CI [-.0134, -.0024], *CSIE* = -.05, and less Perceived Threat, *b* = -.0638, CI [-.0987, -.0370], *CSIE* = -.0942.

Four separate Ingroup-Binding mediations of Political Ideology to Bias, to Negative Bias, to Implicit Bias, and to Threat were conducted. We observed a significant indirect effect of the Ingroup-Binding foundations in which more investment was related to more Bias, *b* = -.0370, CI [-.0725, -.0064], *CSIE* = -.0491, (Ingroup-Binding to Bias *b* = .1890), more Negative Bias, *b* = -.1314, CI [-.1935, -.0839], *CSIE* = -.1193, (Ingroup-Binding to Negative Bias *b* = .6713), more Implicit Bias, *b* = -.0088, CI [-.0150, -.0040], *CSIE* = -.0683 (Ingroup-Binding to Implicit, *b* = -.0436, and more Perceived Threat, *b* = -.0940, CI [-.1325, -.0653], *CSIE* = -.1387, (Ingroup-Binding to Threat *b* = .4802).

**Section 6:**

**Individualizing-Ingroup Preference to Attitude Bias, to Negative Bias, & to Threat regressions, and Binding-Ingroup Preference to Attitude Bias, to Negative Bias, and to Threat regressions.**

**Study 2:**

We conducted six linear regressions to establish the influence of the manipulated mediators on the outcome variables (Spencer et al., 2005). As predicted, we observed that more Individualizing-Ingroup Preference was significantly related to more Attitude Bias toward Pakistani-immigrants, *R^2^* = .19, *ϐ* = .44, *t*(305) = 8.51, *p* < .001, to more Negative Bias, *R^2^* = .25, *ϐ* = .50, *t*(305) = 10.04, *p* <.001, and to more Perceived Threat, *R^2^* = .24, *ϐ* = .50, *t*(305) = 9.80, *p* < .001 with Bonferroni corrections for three tests.

We also observed as predicted that Binding-Ingroup Preference was significantly related to more Attitude Bias toward Pakistani-immigrants, *R^2^* = .32, *ϐ* = .56, *t*(305) = 11.88, *p* < .001, to more Negative Bias, *R^2^* = .31, *ϐ* = .56, *t*(305) = 11.73, *p* <.001, and to more Perceived Threat, *R^2^* = .38, *ϐ* = .61, *t*(305) = 13.58, *p* < .001. See Supplemental **Section 7** for the predicted significant mediations.

**Study 3:**

We again conducted separate linear regressions for the manipulated mediators to demonstrate the effectiveness of the manipulation. As predicted, we observed that more endorsement of Individualizing-Ingroup Preference was significantly related to more Attitude Bias toward Pakistani-immigrants, *R^2^* = .29, *ϐ* = .53, *t*(395) = 12.56, *p* < .001, to more Negative Bias, *R^2^* = .29, *ϐ* = .54, *t*(395) = 12.57, *p* <.001, to more Perceived Threat, *R^2^* = .31, *ϐ* = .56, *t*(395) = 13.38, *p* < .001, and to more Implicit Bias, *R^2^* = .10, *ϐ* = .32, *t*(381) = 6.66, *p* < .001.

As predicted, we also observed that Binding-Ingroup Preference was significantly related to more Attitude Bias toward Pakistani-immigrants, *R^2^* = .26, *ϐ* = .51, *t*(395) = 11.82, *p* < .001, to more Negative Bias, *R^2^* = .26, *ϐ* = .51, *t*(395) = 11.71, *p* <.001, to more Perceived Threat, *R^2^* = .39, *ϐ* = .62, *t*(395) = 15.79, *p* < .001, and to more Implicit Bias, *R^2^* = .12, *ϐ* = .34, *t*(381) = 7.08, *p* < .001. See Supplemental **Section 7** for the predicted significant mediations.

**Section 7:** **Individualizing-Ingroup Preference index and Binding-Ingroup Preference index multiple mediations**.

**Study 2**:

We conducted three Individualizing-Ingroup Preference and Binding-Ingroup Preference multiple mediations of Political Ideology to Bias, to Negative Bias, and to Threat. We observed the predicted significant indirect effect of the Individualizing-Ingroup Preference scores in which more investment was related to more Bias, *b* = -.0278, CI [-.0666, -.0054], *Completely Standardized Indirect Effect (CSIE)* = -.0354, more Negative Bias, *b* = -.0628, CI [-.1245, -.0263], *CSIE* = -.0596, and more Perceived Threat, *b* = -.0291, CI [-.0597, -.0099], *CSIE* = -.0430.

We observed the predicted significant indirect effect of the Binding-Ingroup Preference scores in which more investment was related to more Bias, *b* = -.1202, CI [-.1838, -.0733], *CSIE* = -.1529, more Negative Bias, *b* = -.1367, CI [-.2259, -.0721], *CSIE* = -.1297, and more Perceived Threat, *b* = -.0971, CI [-.1472, -.0608], *CSIE* = -.1436.

**Study 3**:

We conducted four Individualizing-Ingroup Preference and Binding-Ingroup Preference multiple mediations of Political Ideology to Bias, to Negative Bias, to Threat, and to Implicit Bias. We observed the predicted significant indirect effect of the Individualizing-Ingroup Preference scores in which more investment was related to more Bias, *b* = -.0693, CI [-.1141, -.0369], *Completely Standardized Indirect Effect (CSIE)* = -.0920, more Negative Bias, *b* = -.1016, CI [-.1652, -.0533], *CSIE* = -.0923, more Perceived Threat, *b* = -.0398, CI [-.0696, -.0189], *CSIE* = -.0588, and more Implicit Bias, *b* = -.0054, CI [-.0122, -.0012], *CSIE* = -.0417.

We observed the predicted significant indirect effect of the Binding-Ingroup Preference scores in which more investment was related to more Bias, *b* = -.0391, CI [-.0801, -.0013], *CSIE* = -.0519, more Negative Bias, *b* = -.0674, CI [-.1319, -.0112], *CSIE* = -.0611, and more Perceived Threat, *b* = -.0841, CI [-.1239, -.0515], *CSIE* = -.1241; the indirect effect of Implicit Bias was also significant, *b* = -.0071, CI [-.0161, -.0002], *CSIE* = -.0552.

**Separate Individualizing-Ingroup Preference index and Binding-Ingroup Preference index single mediations**.

**Study 2**:

We conducted three Individualizing-Ingroup Preference single mediations of Political Ideology to Bias, to Negative Bias, and to Threat. We observed a significant indirect effect of the Individualizing-Ingroup Preference scores in which more investment was related to more Bias, *b* = -.0686, CI [-.1196, -.0334], *Completely Standardized Indirect Effect (CSIE)* = -.0872, more Negative Bias, *b* = -.1092, CI [-.1847, -.0540], *CSIE* = -.1036, and more Perceived Threat, *b* = -.0620, CI [-.1035, -.0318], *CSIE* = -.0917.

We conducted three Binding-Ingroup Preference mediations of Political Ideology to Bias, to Negative Bias, and to Threat. We observed a significant indirect effect of the Binding-Ingroup Preference scores in which more investment was related to more Bias, *b* = -.1482, CI [-.2153, -.0969], *CSIE* = -.1884, (Binding to Bias *b* = .9392), more Negative Bias, *b* = -.1997, CI [-.3033, -.1213], *CSIE* = -.1894, (Binding to Negative Bias *b* = 1.2657), and more Perceived Threat, *b* = -.1262, CI [-.1798, -.0839], *CSIE* = -.1867, (Binding to Threat *b* = .8001).

**Study 3**:

We conducted four Individualizing-Ingroup Preference single mediations of Political Ideology to Bias, to Negative Bias, to Threat, and to Implicit Bias. We observed a significant indirect effect of the Individualizing-Ingroup Preference scores in which more investment was related to more Bias, *b* = -.0875, CI [-.1299, -.0538], *Completely Standardized Indirect Effect (CSIE)* = -.1161, more Negative Bias, *b* = -.1330, CI [-.2003, -.0811], *CSIE* = -.1208, more Perceived Threat, *b* = -.0790, CI [-.1133, -.0496], *CSIE* = -.1166, and more Implicit Bias, *b* = -.0087, CI [-.0160, -.0042], *CSIE* = -.0671.

We conducted four Binding-Ingroup Preference mediations of Political Ideology to Bias, to Negative Bias, to Threat, and to Implicit Bias. We observed a significant indirect effect of the Binding-Ingroup Preference scores in which more investment was related to more Bias, *b* = -.1183, CI [-.1627, -.0832], *CSIE* = -.1571, (Binding to Bias *b* = .6650), more Negative Bias, *b* = -.1836, CI [-.2578, -.1255], *CSIE* = -.1667, (Binding to Negative Bias *b* = 1.0322), more Perceived Threat, *b* = -.1296, CI [-.1982, -.0969], *CSIE* = -.1913, (Binding to Threat *b* = .7288), and more Implicit Bias, *b* = -.0133, CI [-.0217, -.0071], *CSIE* = -.1031, (Binding to Implicit *b* = .0756).

**Section 8a:** Ingroup Preference regressions for each of the five foundations for Studies 1, 2, & 3

**Section 8b:** Ingroup and Outgroup regressions for each of the five foundations for Studies 1, 2, & 3.

**Study 1**:

We observed that Political Ideology was significantly and negatively correlated with each Preference Score (see below). Bias corrected and accelerated (BCa) bootstrapping of these regression analyses (5000 samples with 95% CI) replicated these results with the exception of harm becoming non-significant.

Standardized regression coefficients (*β*) for regression equations with Framed moral foundation ingroup-preference scores predicting Political Ideology.

|  | Political Ideology | | | | | | |  |
| --- | --- | --- | --- | --- | --- | --- | --- | --- |
|  | | Linear Regressions | | |  | Bootstrapping (BCa) | | |
|  | | ***β*** | ***p-value*** | ***R^2^*** | | ***df*** | ***b*** | ***95% CI for b*** |
| Harm-Ingroup Preference | | -.18* | = .029 | .03 | | 151 | -.06 | [-.126, .002] |
|  | |  |  |  | |  |  |  |
| Fairness-Ingroup Preference | | -.36*** | < .001 | .13 | | 151 | -.14 | [-.201, -.075] |
|  | |  |  |  | |  |  |  |
| Loyalty-Ingroup Preference | | -.36*** | < .001 | .13 | | 151 | -.15 | [-.215, -.086] |
|  | |  |  |  | |  |  |  |
| Authority-Ingroup Preference | | -.37*** | < .001 | .14 | | 151 | -.14 | [-.204, -.087] |
|  | |  |  |  | |  |  |  |
| Purity-Ingroup Preference | | -.33*** | < .001 | .11 | | 151 | -.15 | [-.229, -.077] |

*Note.* Higher scores on ideology reflected increased liberalism (vs. conservatism). Higher Ingroup Preference Scores indicated more investment in the moral foundation when it was framed about the ingroup. A negative regression coefficient between Preference Score and Political Ideology indicated that conservatives showed more endorsement of the moral foundation when it was framed about the ingroup as opposed to the outgroup. Bootstrapping analyses were conducted to 5000 samples.

**Study 2**: **Ingroup Preference regressions for each of the five foundations**

Standardized regression coefficients (*β*) for regression equations with Framed moral foundation preference scores predicted by Political Ideology.

|  | Political Ideology | | | | | | | |  |  |
| --- | --- | --- | --- | --- | --- | --- | --- | --- | --- | --- |
|  | | Linear Regressions | | | Bootstrapping (BCa) | | | | |  |
|  | | ***β*** | ***p-value*** | ***R^2^*** | | ***df*** | ***b*** | ***95% CI for b*** | |  |
| Harm-Ingroup Preference | | -.23*** | < .001 | .05 | | 305 | -.10 | [-.152, -.048] | | |
|  | |  |  |  | |  |  |  | |  |
| Fairness-Ingroup Preference | | -.21*** | < .001 | .05 | | 305 | -.11 | [-.172, -.047] | |  |
|  | |  |  |  | |  |  |  | |  |
| Loyalty-Ingroup Preference | | -.41*** | < .001 | .17 | | 305 | -.20 | [-.250, -.141] | |  |
|  | |  |  |  | |  |  |  | |  |
| Authority-Ingroup Preference | | -.37*** | < .001 | .14 | | 305 | -.17 | [-.228, -.116] | |  |
|  | |  |  |  | |  |  |  | |  |
| Purity-Ingroup Preference | | -.22*** | < .001 | .05 | | 305 | -.11 | [-.164, -.049] | |  |

*Note.* Higher Ingroup Preference Scores indicated more investment in the moral foundation when it was framed about the ingroup. A negative regression coefficient between Preference Score and Political Ideology indicated that conservatives showed more endorsement of the moral foundation when it was framed about the ingroup as opposed to the outgroup.

**Study 3:**

Standardized regression coefficients (*β*) for regression equations with Framed moral foundation ingroup-preference scores predicted by Political Ideology.

|  | Political Ideology | | | | | | |  |
| --- | --- | --- | --- | --- | --- | --- | --- | --- |
|  | | Linear Regressions | | | Bootstrapping (BCa) | | | |
|  | | ***β*** | ***p-value*** | ***R^2^*** | ***df*** | ***b*** | ***95% CI for b*** | |
| Harm-Ingroup Preference | | -.21*** | < .001 | .05 | 395 | -.10 | [-.146, -.052] | |
|  | |  |  |  |  |  |  | |
| Fairness-Ingroup Preference | | -.25*** | < .001 | .06 | 395 | -.13 | [-.182, -.077] | |
|  | |  |  |  |  |  |  | |
| Loyalty-Ingroup Preference | | -.37*** | < .001 | .14 | 395 | -.20 | [-.250, -.146] | |
|  | |  |  |  |  |  |  | |
| Authority-Ingroup Preference | | -.37*** | < .001 | .13 | 395 | -.18 | [-.228, -.135] | |
|  | |  |  |  |  |  |  | |
| Purity-Ingroup Preference | | -.29*** | < .001 | .09 | 395 | -.15 | [-.204, -.101] | |

**Section 8b:** Ingroup and Outgroup regressions for each of the five foundations for Studies 1, 2, & 3.

**Study 1:**

Fourteen linear regressions with Political Ideology entered as predictor and Ingroup and Outgroup moral foundations acting as the outcome variables. Variables were rescored so that they matched the 0 to 5 coding for the Moral Foundations Questionnaire.

|  | Political Ideology | | | | | |  |
| --- | --- | --- | --- | --- | --- | --- | --- |
|  | | Linear Regressions | | |  | Bootstrapping (BCa) | |
| Ingroup Referent | | ***β*** | ***p-value*** | ***R^2^*** | ***df*** | ***b*** | ***95% CI for b*** |
| ingroup-Harm | | -.04 | = .609 | .00 | 151 | -.02 | [-.094, .049] |
|  | |  |  |  |  |  |  |
| ingroup-Fairness | | .07 | = .406 | .01 | 151 | .03 | [-.034, .086] |
|  | |  |  |  |  |  |  |
| ingroup-Loyalty | | -.47*** | < .001 | .22 | 151 | -.25 | [-.324, -.185] |
|  | |  |  |  |  |  |  |
| ingroup-Authority | | -.41*** | < .001 | .17 | 151 | -.22 | [-.293, -.143] |
|  | |  |  |  |  |  |  |
| ingroup-Purity | | -.49*** | < .001 | .24 | 151 | -.32 | [-.416, -.224] |
| Outgroup Referent | |  |  |  |  |  |  |
| outgroup-Harm | | .09 | = .263 | .01 | 151 | .04 | [-.040, .118] |
|  | |  |  |  |  |  |  |
| outgroup-Fairness | | .34*** | < .001 | .11 | 151 | .17 | [.088, .243] |
|  | |  |  |  |  |  |  |
| outgroup-Loyalty | | -.20* | = .014 | .04 | 151 | -.10 | [-.179, -.030] |
|  | |  |  |  |  |  |  |
| outgroup-Authority | | -.14 | = .077 | .02 | 151 | -.07 | [-.156, .011] |
|  | |  |  |  |  |  |  |
| outgroup-Purity | | -.31*** | < .001 | .10 | 151 | -.17 | [-.249, -.087] |

*Note.* Higher scores on ideology reflected increased liberalism (vs. conservatism). A negative regression coefficient between Ingroup- or Outgroup-Score and Political Ideology indicated that conservatives showed more endorsement of the moral foundation. Bootstrapping analyses were conducted to 5000 samples.

**Study 2: Ingroup and Outgroup regressions for each of the five foundations**

Fourteen linear regressions with Political Ideology entered as predictor and Ingroup and Outgroup moral foundations acting as the outcome variables. Variables were rescored so that they matched the 0 to 5 coding for the Moral Foundations Questionnaire.

|  | Political Ideology | | | | | | | | |  | |
| --- | --- | --- | --- | --- | --- | --- | --- | --- | --- | --- | --- |
|  | | Linear Regressions | | | |  | | Bootstrapping (BCa) | | | |
| Ingroup Referent | | | ***β*** | ***p-value*** | ***R^2^*** | | ***df*** | ***b*** | ***95% CI for b*** | |  |
| ingroup-Harm | | | .03 | = .607 | .00 | | 305 | .02 | [-.042, .073] | |  |
|  | | |  |  |  | |  |  |  | |  |
| ingroup-Fairness | | | .12* | = .042 | .01 | | 305 | .06 | [.002, .112] | |  |
|  | | |  |  |  | |  |  |  | |  |
| ingroup-Loyalty | | | -.41*** | < .001 | .17 | | 305 | -.22 | [-.274, -.159] | |  |
|  | | |  |  |  | |  |  |  | |  |
| ingroup-Authority | | | -.42*** | < .001 | .17 | | 305 | -.22 | [-.279, -.163] | |  |
|  | | |  |  |  | |  |  |  | |  |
| ingroup-Purity | | | -.35*** | < .001 | .12 | | 305 | -.20 | [-.260, -.138] | |  |

**Outgroup Referent**

| outgroup-Harm | .22*** | < .001 | .05 | 305 | .12 | [.053, .177] |
| --- | --- | --- | --- | --- | --- | --- |
|  |  |  |  |  |  |  |
| outgroup-Fairness | .31*** | < .001 | .10 | 305 | .17 | [.107, .225] |
|  |  |  |  |  |  |  |
| outgroup-Loyalty | -.05 | = .395 | .00 | 305 | -.02 | [-.068, .025] |
|  |  |  |  |  |  |  |
| outgroup-Authority | -.10 | = .070 | .01 | 305 | -.05 | [-.103, .003] |
|  |  |  |  |  |  |  |
| outgroup-Purity | -.17** | = .003 | .03 | 305 | -.09 | [-.159, -.027] |

Analyses showed that while the Ingroup-Fairness slope was significant alone, it was extremely small, *R^2^* = .01.

**Study 3: Ingroup and Outgroup regressions for each of the five foundations**

For the separate Ingroup-MFQ and Outgroup-MFQ regressions, endorsement of Outgroup-Harm and Outgroup-Fairness was related to more liberalism and less conservatism and endorsement of Ingroup-Loyalty, Ingroup-Authority, and Ingroup-Purity was more strongly related to more conservatism and less liberalism; these results replicated the key findings of Studies 1 and 2. In Study 3, we also did find that Ingroup-Fairness was significant, though the effect size (*R^2^* = .018) was much smaller for this relationship compared to the other variables in Study 3, and was non-significant with Bonferroni correction.

Fourteen linear regressions with Political Ideology entered as predictor and Ingroup and Outgroup moral foundations acting as the outcome variables. Variables were rescored so that they matched the 0 to 5 coding for the Moral Foundations Questionnaire.

|  | Political Ideology | | | | | | |  |
| --- | --- | --- | --- | --- | --- | --- | --- | --- |
|  | | Linear Regressions | | |  | Bootstrapping (BCa) | | |
| Ingroup Referent | | ***β*** | ***p-value*** | ***R^2^*** | ***df*** | ***b*** | ***95% CI for b*** | |
| ingroup-Harm | | .07 | = .161 | .01 | 395 | .04 | [-.021, .092] | |
|  | |  |  |  |  |  |  | |
| ingroup-Fairness | | .13** | = .008 | .02 | 395 | .06 | [.012, .114] | |
|  | |  |  |  |  |  |  | |
| ingroup-Loyalty | | -.35*** | < .001 | .12 | 395 | -.20 | [-.253, -.142] | |
|  | |  |  |  |  |  |  | |
| ingroup-Authority | | -.39*** | < .001 | .15 | 395 | -.21 | [-.263, -.155] | |
|  | |  |  |  |  |  |  | |
| ingroup-Purity | | -.32*** | < .001 | .10 | 395 | -.18 | [-.233, -.127] | |

**Outgroup Referent**

| outgroup-Harm | .25*** | < .001 | .06 | 395 | .14 | [.079, .191] |
| --- | --- | --- | --- | --- | --- | --- |
|  |  |  |  |  |  |  |
| outgroup-Fairness | .34*** | < .001 | .12 | 395 | .19 | [.134, .257] |
|  |  |  |  |  |  |  |
| outgroup-Loyalty | .00 | = .942 | .00 | 395 | .00 | [-.048, .050] |
|  |  |  |  |  |  |  |
| outgroup-Authority | -.06 | = .239 | .00 | 395 | -.03 | [-.077, .020] |
|  |  |  |  |  |  |  |
| outgroup-Purity | -.05 | = .348 | .00 | 395 | -.03 | [-.082, .030] |

**Section 9:**

**Eight orders to which participants were randomly assigned in Study 3.**

| Ingroup MFQ | Filler then Outgroup MFQ | N. cog filler | Bias and Negative Bias | N. cog filler | Threat | AMP |
| --- | --- | --- | --- | --- | --- | --- |
| Ingroup MFQ | Filler then Outgroup MFQ | N. cog filler | Threat | N. cog filler | Bias and Negative Bias | AMP |
| Ingroup MFQ | Filler then Outgroup MFQ | N. cog filler | AMP | N. cog filler | Bias and Negative Bias | Threat |
| Ingroup MFQ | Filler then Outgroup MFQ | N. cog filler | AMP | N. cog filler | Threat | Bias and Negative Bias |
| Outgroup MFQ | Filler then Ingroup MFQ | N. cog filler | Bias and Negative Bias | N. cog filler | Threat | AMP |
| Outgroup MFQ | Filler then Ingroup MFQ | N. cog filler | Threat | N. cog filler | Bias and Negative Bias | AMP |
| Outgroup MFQ | Filler then Ingroup MFQ | N. cog filler | AMP | N. cog filler | Bias and Negative Bias | Threat |
| Outgroup MFQ | Filler then Ingroup MFQ | N. cog filler | AMP | N. cog filler | Threat | Bias and Negative Bias |

AMP = Affect Misattribution Procedure

N. Cog = Need for Cognition four item filler task
